# Critical Bimetallic Phosphide Layer Enables Fast Electron Transfer and Extra Energy Supply for Flexible Quasi-Solid-State Zinc Batteries

**DOI:** 10.1007/s40820-025-01784-3

**Published:** 2025-05-21

**Authors:** Leixin Wu, Linfeng Lv, Yibo Xiong, Wenwu Wang, Xiaoqiao Liao, Xiyao Huang, Ruiqi Song, Zhe Zhu, Yixue Duan, Lei Wang, Zeyu Ma, Jiangwang Wang, Fazal ul Nisa, Kai Yang, Muhammad Tahir, Longbing Qu, Wenlong Cai, Liang He

**Affiliations:** 1 School of Mechanical Engineering, State Key Laboratory of Intelligent Construction and Healthy Operation and Maintenance of Deep Underground Engineering, Sichuan University, Chengdu, 610065 People’s Republic of China; 2 Department of Mechanical Engineering, City University of Hong Kong, Tat Chee Avenue, Hong Kong, 999077 People’s Republic of China; 3 School of Chemistry, Faculty of Science, University of New South Wales, Sydney, NSW 2052 Australia; 4 School of Mechatronical Engineering, Beijing Institute of Technology, Beijing, 100081 People’s Republic of China; 5 Department of Chemical Engineering, The University of Melbourne, Melbourne, VIC 3010 Australia; 6 College of Materials Science and Engineering, Sichuan University, Chengdu, 610064 People’s Republic of China; 7 Med+X Center for Manufacturing, West China Hospital, Sichuan University, Chengdu, 610041 People’s Republic of China; 8 Yibin Industrial Technology Research Institute of Sichuan University, Yibin R&D Park of Sichuan University, Yibin, 644005 People’s Republic of China

**Keywords:** Bimetallic phosphide layer, Dual functionality, Fast electron transfer, Energy supply, Flexible quasi-solid-state batteries

## Abstract

Nickel-based cathodes in aqueous nickel-zinc batteries typically suffer from sluggish reaction kinetics and limited energy density. In situ introduction of metal phosphides and rational construction of heterostructures can effectively promote electron/ion transport. However, the complex evolution of phosphidation and intractable phosphidizing degree greatly affect the composition of active phase, active sites, charge transfer rate, and ion adsorption strength of cathodes. Herein, the critical bimetallic phosphide layer (CBPL) is constructed on the NiCo-layered double hydroxide (NiCo-LDH) skeleton by a controllable anion-exchange strategy, yielding a novel nanohybrid cathode (NiCo-P1.0, 1.0 representing the mass ratio of Na_2_H_2_PO_2_ to NiCo-LDH). The high-conductivity CBPL with the inner NiCo-LDH forms extensive heterostructures, effectively regulating the electronic structure via charge transfer, thereby improving electrical conductivity. Remarkably, the CBPL exhibits unexpected electrochemical activity and synergizes with NiCo-LDH for electrode reactions, ultimately delivering extra energy. Benefiting from the bifunctional CBPL, NiCo-P1.0 delivers an optimal capacity of 286.64 mAh g^−1^ at 1C (1C = 289 mAh g^−1^) and superb rate performance (a capacity retention of 72.22% at 40C). The assembled NiCo-P1.0//Zn battery achieves ultrahigh energy/power density (503.62 Wh kg^−1^/18.62 kW kg^−1^, based on the mass loading of active material on the cathode), and the flexible quasi-solid-state pouch cell validates its practicality. This work demonstrates the superiority of bifunctional CBPL for surface modification, providing an effective and scalable compositing strategy in achieving high-performance cathodes for aqueous batteries.

**Supplementary Information:**

The online version contains supplementary material available at 10.1007/s40820-025-01784-3.

## Highlights

- The critical bimetallic phosphide layer (CBPL) exhibits high electrical conductivity and forms heterostructures with NiCo-layered double hydroxide (NiCo-LDH), improving the electrical conductivity of the hybrid cathode (NiCo-P1.0).
- CBPL facilitates OH⁻ adsorption and synergizes with NiCo-LDH in electrode reactions, delivering extra energy.
- NiCo-P1.0 cathode delivers 286.64 mAh g⁻¹ at 1C with a retention of 72.22% at 40C. The assembled NiCo-P1.0//Zn battery achieves energy density/power density (503.62 Wh kg^−1^/18.62 kW kg^−1^). The flexible quasi-solid-state pouch cell maintains stable output after deformation.

**Supplementary Information:**

The online version contains supplementary material available at 10.1007/s40820-025-01784-3.

## Graphical abstract

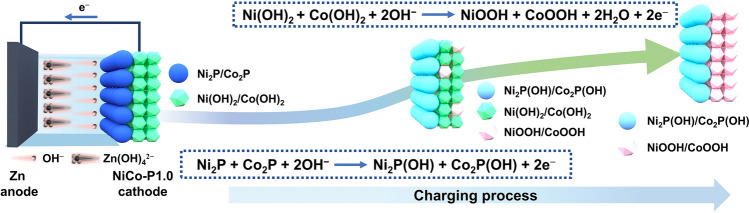

## Introduction

With the gradual depletion of non-renewable energy sources and the inducing environmental crisis, the development of advanced and highly efficient energy storage units is of great significance [1]. Among numerous energy storage devices, aqueous zinc batteries (ZBs) are recognized as one of the most viable options for energy storage owing to their high energy density, intrinsic safety, and simple as well as inexpensive manufacturing process [2, 3]. As a member of the ZBs systems, nickel-zinc batteries (NZBs) have attracted extensive research interest due to their high discharge voltage (over 1.7 V) [4, 5]. There have been many successful studies about improving corrosion and dendrite problems for the zinc anode which mainly affect the safety and stability of batteries [6–8]. However, the nickel-based cathode, largely determining the upper-performance limit of NZBs, suffers from major obstacles: (1) limited energy density caused by the low utilization efficiency of the nickel cathode, (2) slow reaction kinetics due to inherently retarded electrical conductivity and stacking of high impedance by-products from irreversible phase transitions, and (3) capacity degradation induced by structural collapse [9–11]. As a result, the development of NZBs has been severely limited.

To tackle these impediments, researchers have adopted a series of strategies for nickel-based cathodes, containing doping metal atoms, modulating nanostructures, in situ reconfiguration, constructing heterostructures, and surface anion engineering [11–21]. Our research group previously modulated the structure of NiCo-based bimetallic microwires by controlling the cobalt content, which was doped at the atomic scale, thus resulting in a stable crystal structure and higher active surface area [11]. The hierarchical alloy structure achieved dual contribution in terms of area-specific energy and power, effectively solving the inherent performance limitations of monometallic hydroxides. However, this strategy leads to low utilization of the cathode material, with a rate below 20%, resulting in a diminished actual mass capacity. Besides, in situ reconstruction is extensively utilized to expose more active sites, thereby enhancing active material utilization [12, 13, 22]. A Ni–Ni(OH)_2_/Zn(OH)_2_ nanostructured cathode was constructed by Zhu et al. through the reconstruction of nanoporous nickel, and the introduction of an epitaxial Zn(OH)_2_ nanophase [12]. Although the Ni–Zn battery composed of the cathode achieved ultrahigh-rate performance with a capacity retention of 63.8% at a current density 500 times higher, the 10-nm-thick reconstructed activation layer directly indicates the limited utilization of active materials, thereby leading to a constrained energy supply. Additionally, the construction of heterostructure can also improve the reactivity and reaction kinetics of electrode materials [14–16]. Cai et al. reported a MoSe_2_ decorated Ni/Co selenide complex hollow arrayed structures with dense heterointerface [14]. Benefiting from the heterostructure, the cathode demonstrated strong OH^−^ adsorption, a high areal capacity of 1.42 mAh cm^−2^ (corresponding to 167.0 mAh g^−1^) at 2 mA cm^−2^, and a high rate performance (a capacity retention of 85% at 20 mA cm^−2^). However, due to the high proportion of inactive components in the heterostructure, the cathode has limited reaction depth, restricting the energy supply. Furthermore, surface anion engineering is regarded as a pivotal approach to accelerate and deepen electrochemical reactions, thereby enhancing the capacity and rate performance [17, 18]. The hollow NiS-coated Ni_0.95_Zn_0.05_(OH)_2_ microsphere was synthesized by Zhou et al. utilizing low-temperature co-precipitation and anion surface exchange-based Kirkendall effect [17]. Although the hollow microstructure endowed the electrode with ultrahigh tap density and absolute energy supply, the rate performance can only be improved to 5.6 A g^−1^ by the nickel sulfide coating, which still couldn’t meet the current fast charging needs. While the above strategies are effective in certain aspects (more details are summarized in Table S1), they do not fully address the major obstacles faced by nickel-based cathode. Thus, the development of high-conductivity and high-utilization nickel-based cathodes is still quite challenging.

Due to metal phosphides with excellent electrical conductivity derived from high electron delocalization, the phosphidizing strategy has been widely used in the surface treatment of nickel-based electrodes to improve electrode conductivity and accelerate reaction kinetics [9, 23–27]. By controlling the low-temperature polymetallic phosphide generation rate, Zhou et al. enhanced the conductivity of the hollow nanostructured metal-organic frame composite [9]. Although some metal phosphides and phosphates, exhibiting electrochemical activity, are increasingly used as pseudocapacitive electrode materials in supercapacitors [28–33], most reports directly use metal phosphides or phosphates as the only active material. Furthermore, little research indicates that the phosphidizing surface treatment can simultaneously improve rate performance and provide a capacity contribution. This is mainly because the intractable phosphidizing process and the complex phosphidizing products hinder the uniformity and stability of the active phosphides and phosphates [23, 34–40]. Chen et al. reported the evolution process of metal oxides to metals, metal phosphides, and metal phosphates with increasing phosphidation temperature, time, and phosphorus source [39]. Wu et al. reported the transformation from Ni(OH)_2_ to Ni and Ni_2_P during the phosphidizing. Therefore, to fully utilize active metal phosphides or phosphates, a strictly controlled phosphidizing process with a clear product is essential [40]. Additionally, the reaction of active metal phosphides and metal phosphates relies on surface adsorption [26–31, 41, 42], which may limit the reaction depth and make it difficult to synergize with other active materials, ultimately wasting their reactivity. Therefore, the rational construction of composite electrodes that effectively harness the high conductivity and reactivity of metal phosphides, while simultaneously activating and promoting the synergistic interactions with other active materials, is of paramount importance.

Herein, we propose a novel optimal bifunctional surface modification, the critical bimetallic phosphide layer (CBPL), by a gradient phosphidizing treatment on the NiCo-layered double hydroxide (NiCo-LDH) framework. The CBPL exhibits high conductivity and forms extensive heterostructures with inner NiCo-LDH, effectively facilitating electron transport, and enhancing the kinetics of redox reactions. Moreover, the CBPL not only performs as the active site for OH^−^ adsorption, promoting ion transport but synergizes with NiCo-LDH to participate in the electrode reactions, ultimately delivering extra energy. Therefore, due to bifunctional CBPL, the nanohybrid cathode with CBPL (NiCo-P1.0) exhibits optimal performance with a high capacity of 286.64 mAh g^−1^ at 1C (1C = 289 mAh g^−1^) and superb rate performance (a capacity retention of 72.22% at 40C). The assembled NiCo-P1.0//Zn battery achieves excellent energy density/power density (503.62 Wh kg^−1^/18.62 kW kg^−1^). In addition, the utility of the NiCo-P1.0 electrode is proved in the flexible quasi-solid-state pouch cell, and its output performance is not affected after cell deformation. This work reveals the dual functionality of surface-modified layers, providing valuable references for developing high-performance cathode materials.

## Experimental Section

### Chemical Reagents

All of the reagents used in this experiment were analytical grade and used without further purification. Nickel nitrate hexahydrate (Ni(NO_3_)_2_·6H_2_O), acrylamide (AM), N, N’ methylene diacrylamide, ammonium persulfate (APS), and N, N, N’, N’-tetramethyl-ethylenediamine (TMED) were purchased from Chengdu Kelong Chemical Co. Ltd. Cobalt nitrate hexahydrate (Co(NO_3_)_2_·6H_2_O), sodium citrate (Na_3_C_6_H_5_O_7_), potassium hydroxide (KOH), and ammonia monohydrate (NH_3_·H_2_O) were purchased from Chengdu Zhuo Pu Instrument Co. Ltd. Sodium monophosphate (NaH_2_PO_2_) and zinc oxide (ZnO) powder were purchased from Chengdu Dingsheng Times Technology Co. Ltd. The zinc (Zn) foil (thickness: 100 μm), carbon cloth, and titanium (Ti) foil (thickness: 30 μm) were provided by Canrd New Energy Technology 62 Co. Ltd.

### Synthesis of Ni(OH)_2_

Ni(OH)_2_ was synthesized via a co-precipitation method. A solution containing 50 mM Ni(NO_3_)_2_·6H_2_O, 0.1 M Na_3_C_6_H_5_O_7_, 0.2 M KOH, and 0.5 M NH_3_·H_2_O in 100 mL Milli-Q water (> 18 MΩ cm) was stirred at 50 °C for 10 h. Afterward, the supernatant was decanted, and the viscous residue was washed with Milli-Q water and then dried at 90 °C overnight to obtain Ni(OH)_2_.

### Synthesis of NiCo-LDH

NiCo-LDH was synthesized using the same procedures as Ni(OH)_2_, with the addition of 12.5 mM Co(NO_3_)_2_·6H_2_O into the solution.

### Synthesis of NiCo-P*x*

NaH_2_PO_2_ and NiCo-LDH were placed in two porcelain boats with mass ratios of NaH_2_PO_2_ to NiCo-LDH at 0, 0.5, 1.0, 1.5, 2.0, and 3.0, respectively. In the tubular furnace, a ceramic boat containing 1 g of NiCo-LDH was placed under downstream of the gas flow, and another one containing the corresponding mass of NaH_2_PO_2_ (based on the specific mass ratios) was placed upstream of the gas flow. Under the Ar atmosphere, NiCo-P*x* (*x* = 0, 0.5, 1.0, 1.5, 2.0, 3.0, representing the mass ratio of Na_2_H_2_PO_2_ to NiCo-LDH) was obtained by maintaining the temperature at 350 °C for 3 h with a heating rate of 1 °C min^−1^.

### Preparation of Ni(OH)_2_, NiCo-LDH, and NiCo-P***x*** Cathode

All the cathodes were synthesized by mixing active materials, carbon black, and polyvinylidene difluoride (PVDF) at a weight ratio of 8: 1: 1 in n-methyl pyrrolidone (NMP). Then, the obtained slurry was coated onto the flexible carbon cloth and dried in a vacuum oven at 60 °C for 12 h. The average mass loading of active materials was 1.8–2.2 mg cm^−2^ (Table S2).

### Preparation of Zn Anode of NiCo-P1.0//Zn Battery

The Zn anode was obtained by electrodepositing Zn onto the carbon cloth at a constant voltage of − 3 V in a three-electrode system, in which the carbon cloth was used as the working electrode, Hg/HgO as the reference electrode, Pt plate as the counter electrode, and a 3 M KOH solution with saturated ZnO was employed as the electrolyte. The average mass loading of Zn was 0.05 g cm^−2^.

### Preparation of Hydrogel Electrolyte of the Flexible Pouch Cells

Three grams of AM and 0.0015 g of N, N’ methylene diacrylamide were dispersed in 10 mL deionized (DI) water under ultrasonication. Afterward, 0.01 g of APS initiators and 8 μL of TMED catalysts were added into the above solution to initiate polymerization under stirring. Subsequently, the mixed solution was quickly poured into the container and then reacted at 50 °C for 24 h to obtain the hydrogel electrolyte. Then, the hydrogel electrolyte was immersed in 3 M KOH solution with saturated ZnO for 24 h for subsequent applications (the synthesis method of hydrogel electrolyte learned from Qu group’s work [43]).

## Results and Discussion

**Fig. 1:**
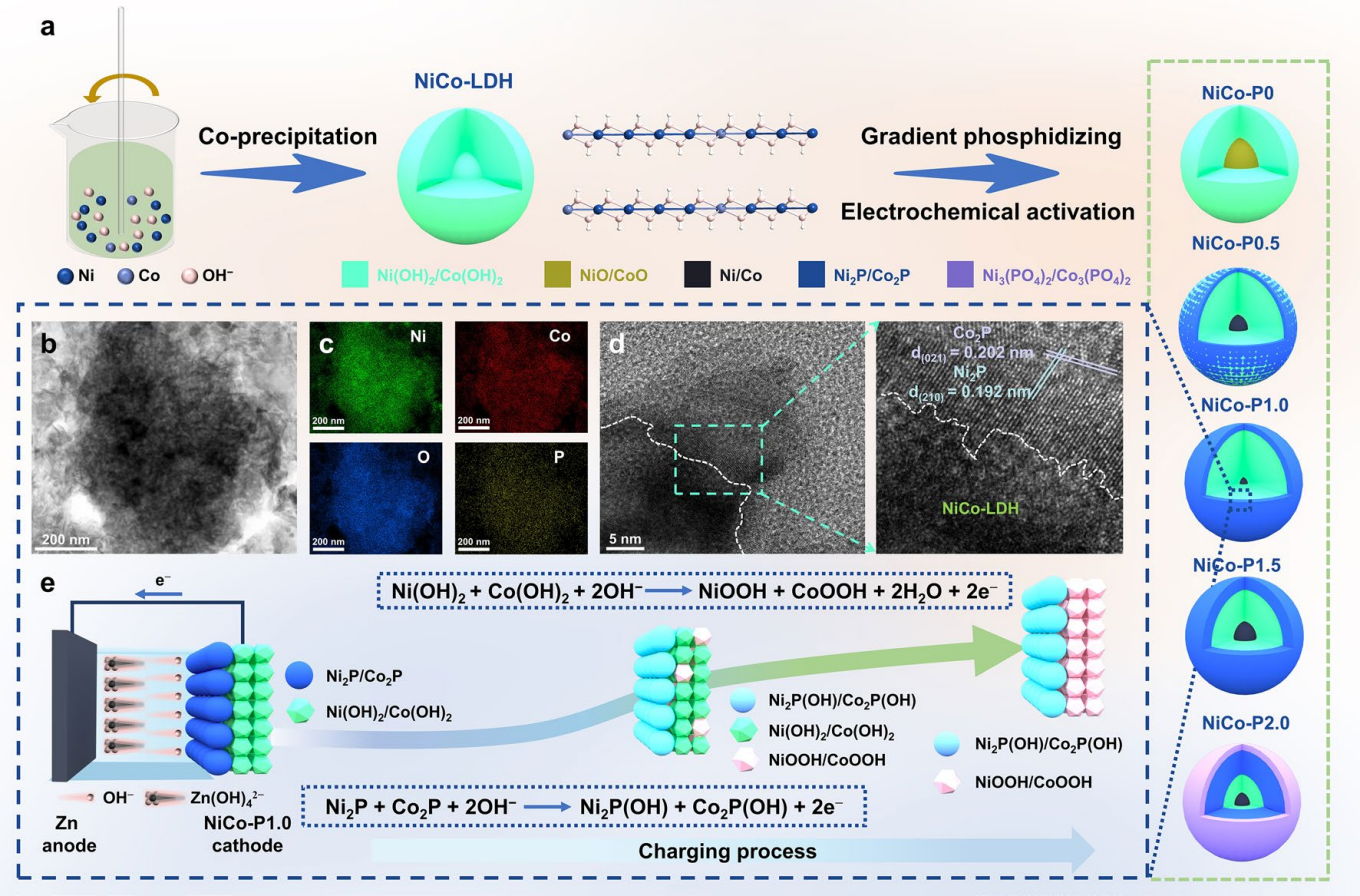
**a** Illustrations for the synthesis of NiCo-P1.0, and the structure of NiCo-LDH and NiCo-P*x* (when *x* = 0, 0.5, 1.0, 1.5, 2.0). **b, c** TEM image, the corresponding EDS mapping, and **d** HRTEM images of NiCo-P1.0. **e** Schematic of NiCo-P1.0//Zn battery and the structure evolution of NiCo-P1.0 during the charging process

The fabrication process of obtaining NiCo-P1.0 is shown in Fig. 1a. Ni(OH)_2_ and NiCo-LDH are first synthesized using a co-precipitation method. Subsequently, after a gradient phosphidizing treatment and electrochemical activation (EA), NiCo-LDH is transformed into electrode materials with different degrees of bimetallic phosphide layer (BPL), denoted as NiCo-P*x* (*x* = 0, 0.5, 1.0, 1.5, 2.0, 3.0). The structure and morphology of NiCo-P1.0 are analyzed using transmission electron microscopy (TEM) and high-resolution TEM (HRTEM). As shown in Fig. 1b, d, NiCo-P1.0 exhibits morphological features resembling a core–shell structure, with the shell being CBPL composed of Ni_2_P/Co_2_P and the core being NiCo-LDH. The corresponding energy dispersive spectroscopy (EDS) mapping (Fig. 1c) demonstrates a uniform distribution of Ni, Co, O, and P elements among NiCo-P1.0. Notably, Fig. 1d directly verifies the existence of the heterostructure, where the (210) lattice fringes of Ni_2_P, the (021) lattice fringes of Co_2_P, and the interface with NiCo-LDH can be distinctly observed. The heterostructure can customize the electronic structure and greatly accelerate the electron transfer, resulting in a deep degree of reactivity and fast reaction kinetics. The schematic for NiCo-P1.0//Zn battery using NiCo-P1.0 as the cathode, 3 M KOH with saturated ZnO solution as the electrolyte, and Zn obtained by electrodeposition as the anode is displayed in Fig. 1e. The working mechanism of the NiCo-P1.0//Zn battery is described in the following reactions:

- Cathode:
  1 $$Ni(OH{)}_{2}+Co(OH{)}_{2}+2{OH}^{-}\leftrightarrow NiOOH+{CoOOH+2H}_{2}O+{2e}^{-}$$
  2 $${Ni}_{2}P+{{Co}_{2}P+2OH}^{-}\leftrightarrow {Ni}_{2}P(OH)+{Co}_{2}P(OH)+2{e}^{-}$$
- Anode:
  3 $$Zn(OH{)}_{4}^{2-}+2{e}^{-}\leftrightarrow Zn+4{OH}^{-}$$
- Overall:
  4 $$\begin{aligned} 2K_{2} [Zn(OH)_{4} ] + & Ni(OH)_{2} + Co(OH)_{2} + Ni_{2} P + Co_{2} P \leftrightarrow 2Zn + NiOOH \\ & + CoOOH + Ni_{2} P(OH) + Co_{2} P(OH) + 4KOH + 2H_{2} O \\ \end{aligned}$$

Notably, the structural evolution of NiCo-P1.0 during charging is illustrated in Fig. 1e. The CBPL reacts at the lower voltage to form Ni_2_P(OH)/Co_2_P(OH), while Ni(OH)_2_/Co(OH)_2_ primarily reacts at the higher voltage to produce NiOOH/CoOOH.

**Fig. 2:**
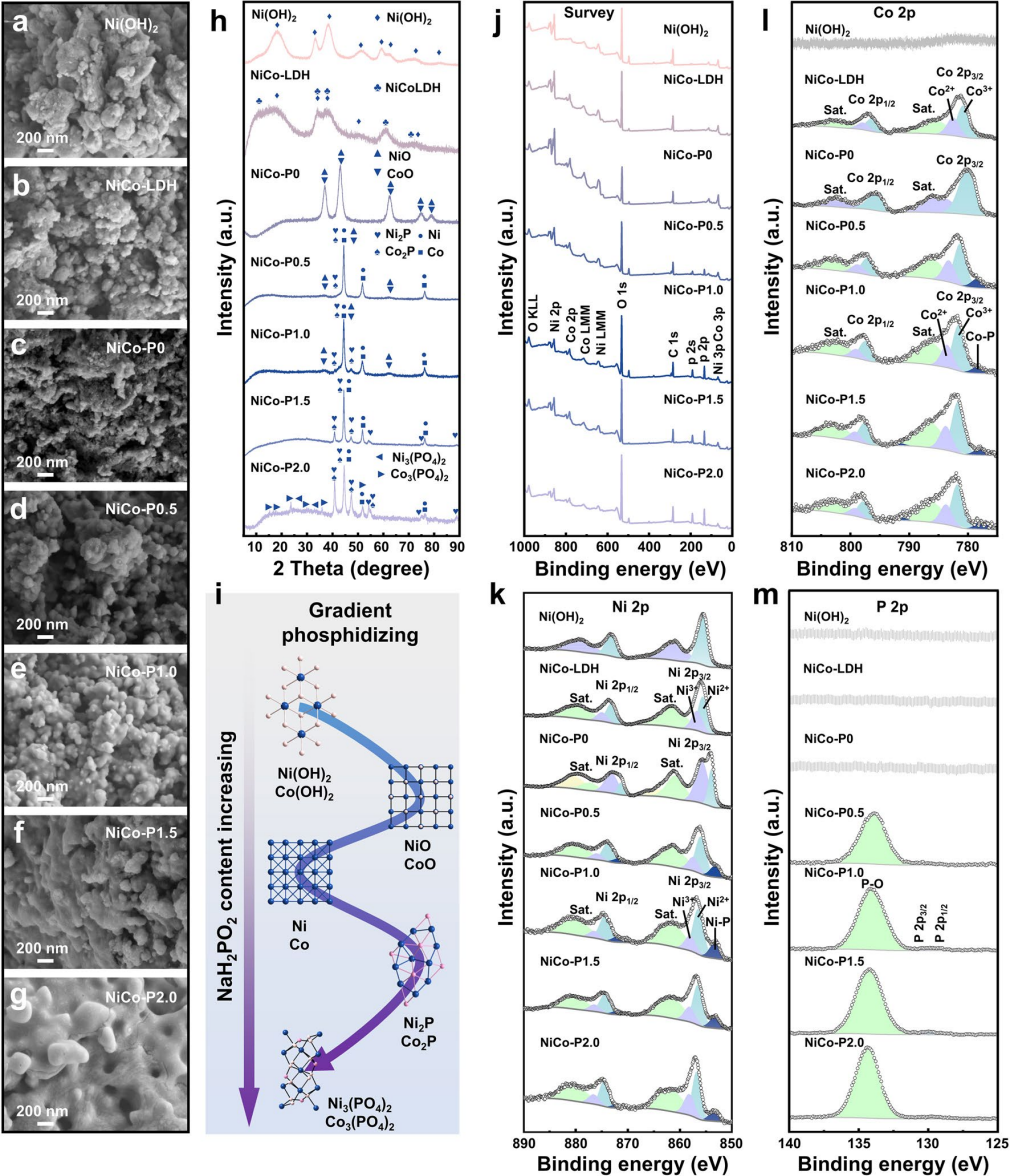
Evolution of the NiCo-LDH during the gradient phosphidizing process. SEM images of **a** Ni(OH)_2_, **b** NiCo-LDH, **c** NiCo-P0, **d** NiCo-P0.5, **e** NiCo-P1.0, **f** NiCo-P1.5, and **g** NiCo-P2.0. **h** XRD patterns of the samples. **i** Schematic of the gradient phosphidizing process of NiCo-LDH with NaH_2_PO_2_’s content increasing. **j** XPS survey spectra and high-resolution XPS spectra of **k** Ni 2*p*, **l** Co 2*p*, and **m** P 2*p* of the samples

Since varying phosphidizing degrees can significantly influence the structure, morphology, and even the composition of the electrode material [23, 34–40], it is essential to focus on the evolution of NiCo-LDH during the gradient phosphidizing process. The structure and morphology of Ni(OH)_2_, NiCo-LDH, and NiCo-P*x* are characterized by scanning electron microscope (SEM) (Figs. 2a-g and S1). Ni(OH)_2_ exhibits the structure of bulky and agglomerated particles, indicating the small active specific surface area and few active sites. Compared with Ni(OH)_2_, NiCo-LDH shows a structure with smaller and more dispersed particles, suggesting that the introduction of the Co element improves the structure. Distinguished from NiCo-P0 possessing a smaller and more dispersed particle structure than NiCo-LDH, the NiCo-P*x* (when *x* = 0.5, 1.0, 1.5, 2.0, 3.0) consists of encapsulated particles. Moreover, the encapsulation worsens and even the surface is blocked as the phosphidizing degree increases, revealing that different degrees of phosphidizing have a significant and direct effect on the surface. In addition, it can be concluded that the specific surface area of NiCo-P*x* decreases linearly with phosphidizing degree increasing, and even the specific surface areas of NiCo-P1.5 and NiCo-P2.0 are less than that of NiCo-LDH (Fig. S2). This suggests that compared with NiCo-LDH, moderate phosphidizing increases the specific surface area, potentially increasing active sites, whereas excessive phosphidizing decreases the specific surface area, which is detrimental to the increase in active sites.

Powder X-ray diffraction (XRD) measurements of Ni(OH)_2_, NiCo-LDH, and NiCo-P*x* are performed to study the crystal structure’s evolution during the gradient phosphidizing process (Figs. 2h and S3). The XRD pattern of Ni(OH)_2_ can be indexed to the hexagonal Ni(OH)_2_ (PDF#57–0907). Since the content ratio of Ni to Co is 4.0, the XRD pattern of NiCo-LDH indexes to hexagonal Ni(OH)_2_ (PDF#57–0907) and hexagonal NiCo-LDH (PDF#40–0216). The XRD result of NiCo-P0 shows that NiCo-LDH dehydrates and transforms into NiO and CoO when only heated without phosphides being introduced. As the phosphidizing degree rises, NiCo-P0.5, NiCo-P1.0, and NiCo-P1.5 are mainly composed of Ni/Co, reduced from Ni(OH)_2_ and NiCo-LDH, with the proportion of Ni_2_P/Co_2_P increasing. At a higher phosphidizing degree, NiCo-P2.0 predominantly consists of Ni_2_P/Co_2_P, accompanied by a small amount of Ni/Co and newly formed Ni_3_(PO_4_)_2_/Co_3_(PO_4_)_2_. With further phosphidizing, NiCo-P3.0 contains Ni_2_P/Co_2_P, Ni_3_(PO_4_)_2_/Co_3_(PO_4_)_2_, and Ni(PO_3_)_2_/Co(PO_3_)_2_. These results suggest that mild phosphidizing produces Ni_2_P/Co_2_P, while extensive phosphidizing leads to the formation of nickel phosphates and cobalt phosphates. Therefore, as the NaH_2_PO_2_ content increases during phosphidizing, the material transitions from Ni(OH)_2_/Co(OH)_2_ to Ni/Co, Ni_2_P/Co_2_P, Ni_3_(PO_4_)_2_/Co_3_(PO_4_)_2_, and Ni(PO_3_)_2_/Co(PO_3_)_2_ (Fig. 2i).

Using X-ray photoelectron spectroscopy (XPS), the surface chemical compositions and valence states of the synthesized catalysts during the gradient phosphidizing process are analyzed. Figures 2j and S4a clearly illustrate the presence of Ni and O elements in Ni(OH)_2_, Ni, Co, and O elements in NiCo-LDH and NiCo-P0, as well as Ni, Co, O, and P elements in NiCo-P*x* (when *x* = 0.5, 1.0, 1.5, 2.0, 3.0). The detailed high-resolution Ni 2*p* spectra of the samples are shown in Figs. 2k and S4b. In the high-resolution Ni 2*p* spectrum of Ni(OH)_2_, the peaks at 855.68 and 873.29 eV correspond to Ni 2*p*_3/2_ and Ni 2p_1/2_, respectively [44]. The Ni 2*p* XPS spectrum of NiCo-LDH shows distinct binding energy peaks at 855.61 and 856.73 eV, corresponding to Ni^2+^ and Ni^3+^, indicating the presence of Ni^3+^ in NiCo-LDH [45, 46]. In the high-resolution Ni 2*p* spectrum of NiCo-P0, the peaks located at 854.12 and 856.08 eV correspond to Ni 2*p*_3/2_, while the peaks at 871.5 and 873.39 eV correspond to Ni 2*p*_1/2_ [47, 48]. The Ni 2*p* XPS spectra of NiCo-P*x* (when *x* = 0.5, 1.0, 1.5, 2.0, 3.0) reveal distinct binding energy peaks at about 856.47, 857.95, and 853.31 eV, corresponding to Ni^2+^, Ni^3+^, and Ni–P, suggesting the introduction of the nickel phosphide  [9, 41, 42, 49]. Similarly, the detailed high-resolution Co 2*p* spectra of the samples are shown in Figs. 2l and S4c. In the high-resolution Co 2*p* spectrum of Ni(OH)_2_, no distinct binding energy peaks are observed, demonstrating the absence of the Co element. The Co 2*p* XPS spectrum of NiCo-LDH shows distinct binding energy peaks at 782.52 and 780.82 eV, corresponding to Co^2+^ and Co^3+^, revealing the presence of Co^3+^ in NiCo-LDH [50]. In the high-resolution Co 2*p* spectrum of NiCo-P0, the peaks located at 780.31 eV correspond to Co 2*p*_3/2_, while the peaks at 795.90 eV correspond to Co 2*p*_1/2_ [51]. The Co 2*p* XPS spectra of NiCo-P*x* (when *x* =0.5, 1.0, 1.5, 2.0, 3.0) reveal distinct binding energy peaks at about 783.65, 781.63, and 778.4 eV, corresponding to Co^2+^, Co^3+^, and Co-P, suggesting the introduction of the cobalt phosphide [9, 41, 49]. The detailed high-resolution P 2*p* spectra of the samples are shown in Figs. 2m and S4d. In the high-resolution P 2*p* spectra of Ni(OH)_2_, NiCo-LDH, and NiCo-P0, no distinct binding energy peaks are observed, demonstrating the absence of the P element. The P 2*p* XPS spectra of NiCo-P0.5, NiCo-P1.0, and NiCo-P1.5 exhibit distinct binding energy peaks at 129.5 and 130 eV, corresponding to P 2*p*_3/2_ and P 2*p*_1/2_, attributed to Ni_2_P/Co_2_P. Notably, the binding energy peak around 134.1 eV, corresponding to phosphate ions, suggests the oxidation of surface phosphides [9, 41, 42, 49, 52]. This also explains the presence of Ni^3+^ in the Ni 2*p* spectra and Co^3+^ in the Co 2*p* spectra of NiCo-P0.5, NiCo-P1.0 and NiCo-P1.5. Furthermore, the P 2*p* XPS spectra of NiCo-P2.0 and NiCo-P3.0 show both phosphate ions and faint metal phosphide peaks, further supporting the formation of phosphates at high phosphidizing levels, in agreement with the XRD results.

The morphology and structure of NiCo-P1.0 are further analyzed using TEM and HRTEM. As shown in Fig. S5a-e, the presence of Ni, Co, P, and O elements can be observed. In Fig. S5f, two sets of lattice fringes are observed. The lattice spacings of 0.204 and 0.177 nm in the core region correspond to the (111) crystal plane of the Ni phase and the (200) crystal plane of the Co phase, respectively. Meanwhile, the lattice spacings of 0.192 and 0.188 nm in the shell region correspond to the (210) crystal plane of the Ni_2_P phase and the (120) crystal plane of the Co_2_P phase, respectively. This structure demonstrates that the CBPL consisting of Ni_2_P/Co_2_P is constructed on the material's surface.

According to experimental discoveries and previous reports [9, 35, 39, 40], the chemical reactions that may occur in the phosphidizing process are Eqs. S9-S18. Furthermore, the phosphidizing of NiCo-LDH with NaH_2_PO_2_ forms a core–shell-like structure, where the shell is BPL consisting of Ni_2_P/Co_2_P. Notably, BPL continuously increases and deepens with increasing phosphidizing degree, manifested by the gradual decrease of the specific surface area and an increase of Ni_2_P/Co_2_P content. When the phosphidizing degree is relatively high, nickel phosphates and cobalt phosphates appear in the phosphidizing layer.

Consistent with previous representative studies on nickel-based cathodes [11, 12, 17, 53–57], the Ni(OH)_2_, NiCo-LDH, and NiCo-P*x* cathodes underwent EA before electrochemical measurements. After EA, NiCo-P1.0 remains its encapsulated particulate morphology (Fig. S6). However, compared with its state before EA (Fig. 2e), the interior of the particles transforms into a nanosheet structure, indicating an increased presence of hydroxides within the sample. The XRD pattern of NiCo-P1.0 after EA (Fig. S7) exhibits characteristic peaks corresponding to Ni(OH)_2_ and Co(OH)_2_, attributed to the activation. As shown in Figs. 1b, d, and S8, NiCo-P1.0 exhibits morphological features resembling a core–shell structure, with the shell being CBPL composed of Ni_2_P/Co_2_P and the core being NiCo-LDH. Besides, the different crystal planes of Ni_2_P, Co_2_P, 4 [Ni(OH)_2_-NiOOH], Ni(OH)_2_, Co(OH)_2_, CoOOH, Ni, and Co in NiCo-P1.0 can also be clearly identified. Notably, the presence of 4 [Ni(OH)_2_-NiOOH] and CoOOH on the surface reveals surface oxidation. The surface chemical composition and valence states of NiCo-P1.0 are evaluated using XPS (Fig. S9). Notably, the signals related to the P element in the XPS spectra are weakened after EA. Since XPS only provides surface information, this signal weakening can be attributed to surface oxidation during EA [42, 52, 55, 58].

To explore the effect of different degrees of BPL on the electrochemical performance, Ni(OH)_2_, NiCo-LDH, and NiCo-P*x* electrodes are evaluated in a three-electrode system. It is worth mentioning that most of the cathodes in NZBs use nickel foam as the current collector. However, previous studies claimed that the nickel foam itself can further transform into reactive hydroxide during the cyclic process [12, 22], so it is difficult to define the actual capacity contribution of active electrode materials. Therefore, the carbon cloth is used as the current collector for Ni(OH)_2_, NiCo-LDH, and NiCo-P*x* cathodes, enabling precise evaluation of the actual capacity contribution of active materials.

**Fig. 3:**
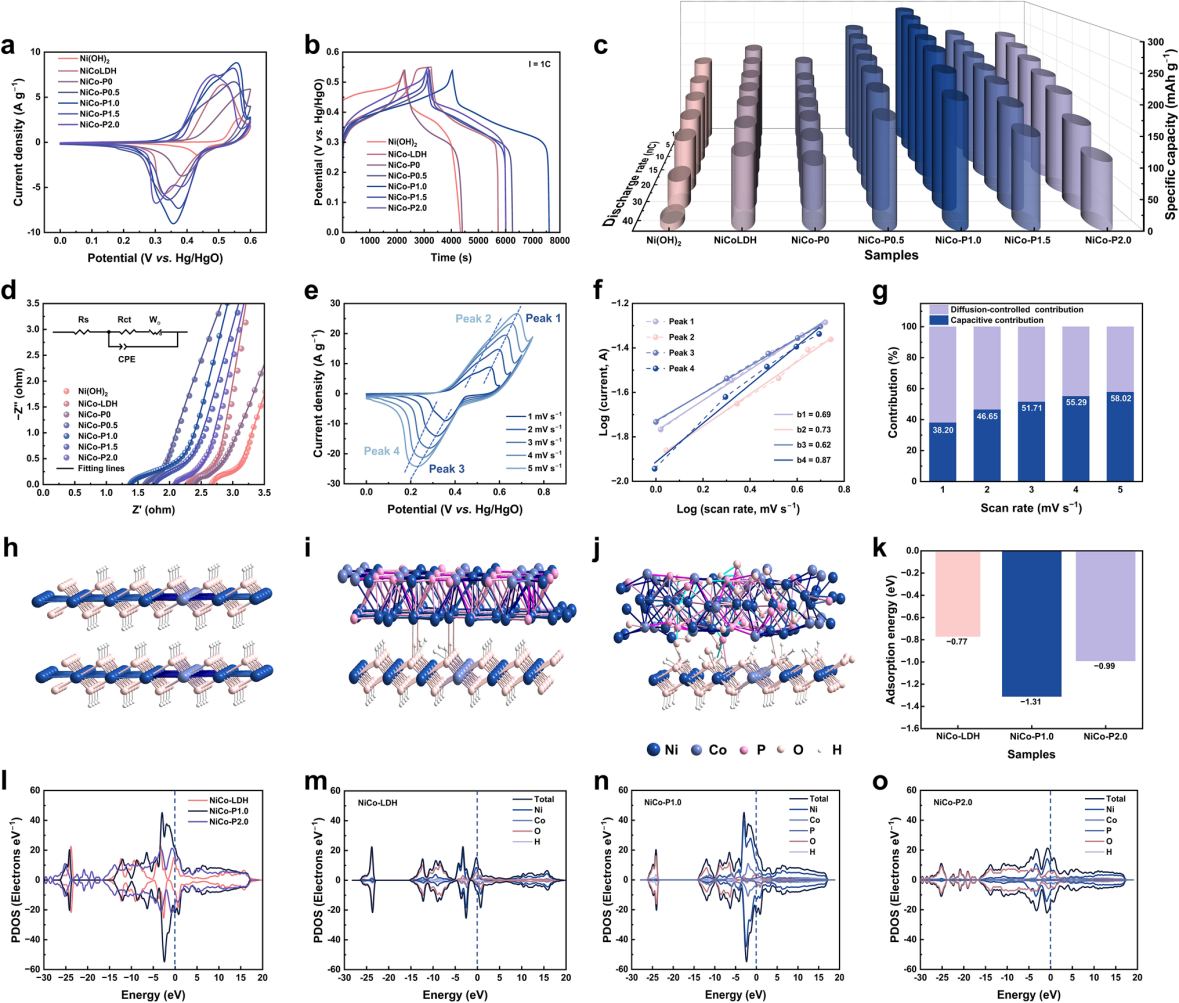
Electrochemical performances of Ni(OH)_2_, NiCo-LDH, and NiCo-P*x* (when *x* = 0, 0.5, 1.0, 1.5, 2.0) in the three-electrode system. **a** CV curves at a scan rate of 1 mV s^−1^. **b** GCD curves of Ni(OH)_2_, NiCo-LDH, and NiCo-P*x* (when *x* = 0, 0.5, 1.0, 1.5, 2.0) at 1C. **c** Comparison of specific capacities. **d** Nyquist plots of Ni(OH)_2_, NiCo-LDH, and NiCo-P*x* (when *x* = 0, 0.5, 1.0, 1.5, 2.0). **e** CV curves of NiCo-P1.0 at various scan rates. **f** The peak currents of NiCo-P1.0 against the scan rates. **g** The calculated capacitive contribution of the NiCo-P1.0 at different scan rates. Optimized atomic structure models of **h** NiCo-LDH, **i** NiCo-P1.0, and **j** NiCo-P2.0. **k** Adsorption energies of OH^−^. **l** DOS of NiCo-LDH, NiCo-P1.0, and NiCo-P2.0. Partial and total DOS of **m** NiCo-LDH, **n** NiCo-P1.0, and **o** NiCo-P2.0

Figures 3a and S10 present the cyclic voltammetry (CV) curves of Ni(OH)_2_, NiCo-LDH, and NiCo-P*x* (when *x* = 0, 0.5, 1.0, 1.5, 2.0) at the scan rate of 1 mV s^−1^ in a potential range from 0 to 0.6 V (*vs.* Hg/HgO). All the CV curves exhibit distinct redox peaks, indicating good electrochemical reversibility. Remarkably, the CV curves of NiCo-P*x* (when *x* = 0.5, 1.0, 1.5, 2.0) show two pairs of redox peaks, distinguishing from the CV curves of Ni(OH)_2_, NiCo-LDH and NiCo-P0 which each show only a single pair of redox peaks (Figs. 3a, e, and S10). Furthermore, the intensity of the redox peak at the lower potential is enhanced as the phosphidizing increases. This suggests that BPL brings a new electrode reaction at the lower potential. Moreover, for further exploring the effect of BPL on the electrode reaction, the charge–discharge behaviors of Ni(OH)_2_, NiCo-LDH, and NiCo-P*x* (when *x* = 0, 0.5, 1.0, 1.5, 2.0) cathodes are analyzed. The galvanostatic charge/discharge (GCD) profiles of all cathodes at different current densities are given in Figs. 3b and S11. Notably, unlike the discharge curves of Ni(OH)_2_, NiCo-LDH and NiCo-P0 at 1C, those of NiCo-P*x* (when *x* = 0.5, 1.0, 1.5, 2.0) have an obvious “potential inflection point” around 0.35 V, apparently related to BPL. Therefore, according to these discussions and the structure of NiCo-P*x*, BPL induces a new electrode reaction at the relatively low potential and supplies extra energy, demonstrating its reactivity and likely synergistic effect with inner NiCo-LDH.

Moreover, the effect of different degrees of BPL on the capacities of cathodes can also be seen based on CV curves (Fig. 3a). NiCo-LDH exhibits a larger curve area (higher capacity) than Ni(OH)_2_, mainly attributed to the intrinsic synergistic effect of bimetallic hydroxides and the exposure of more active sites [59]. Compared with NiCo-P0 with a lower capacity than NiCo-LDH, the capacities of NiCo-P*x* (when *x* = 0.5, 1.0, 1.5, 2.0) are much higher than that of NiCo-LDH, indicating that BPL is favorable to the enhancement of the capacity. Notably, NiCo-P1.0 demonstrates the largest capacity, suggesting that the capacity of NiCo-P*x* does not increase linearly with the increase of BPL. Instead, CBPL exists and leads to the optimal capacity performance of NiCo-P1.0.

By measuring the capacities of various cathodes under varying current densities (based on Eq.S1), the influence of different degrees of BPL on electrochemical performance is further investigated (Fig. 3c and Table S3). Evidently, NiCo-LDH exhibits better capacity and rate performance than Ni(OH)_2_, primarily due to the intrinsic synergistic effects of the bimetallic hydroxide and the increased exposure of active sites [59]. In comparison with NiCo-LDH, NiCo-P0 shows reduced capacity but slightly improved rate performance, indicating that merely heating without introducing BPL does not significantly enhance the electrochemical performance. In contrast, NiCo-P*x* (when *x* = 0.5, 1.0, 1.5, 2.0) exhibits significantly superior capacity and rate performance compared with NiCo-LDH, which is obviously attributed to BPL. Notably, NiCo-P1.0 exhibits the best electrochemical performance, achieving a capacity of 286.64 mAh g^−1^ at 1C, and retaining 207.00 mAh g^−1^ at 40C (a capacity retention of 72.22% at 40C). Therefore, BPL substantially enhances the capacity and rate performance of the cathodes while CBPL leads to the optimal performance of NiCo-P1.0.

The Ohm resistance (R_ohm_) was measured by electrochemical impedance spectroscopy (EIS). The Nyquist plots (Fig. 3d) of Ni(OH)_2_, NiCo-LDH, and NiCo-P*x* demonstrate that the R_ohm_ values of NiCo-P*x* (when *x* = 0.5, 1.0, 1.5, 2.0) are less than that of NiCo-LDH, with NiCo-P1.0 having the smallest R_ohm_. This trend of resistance variation also demonstrates that BPL contributes to the enhancement of the electrical conductivity of cathodes, and CBPL results in optimal electrical conductivity.

Based on the structure and electrochemical behavior of NiCo-P*x*, the overall improvement in the electrochemical performance is attributed to BPL. The active BPL synergistically interacts with the inner NiCo-LDH, causing the NiCo-P*x* (when *x* = 0.5, 1.0, 1.5, 2.0) cathode to undergo two electrode reactions: the Ni(OH)_2_/Co(OH)_2_ electrode reaction at high potential and Ni_2_P/Co_2_P electrode reaction at low potential. Furthermore, the heterostructure formed by active BPL and NiCo-LDH enhances ion transport and facilitates significant synergistic interactions between the active materials, further accelerating and deepening the electrode reactions. Therefore, the capacity of NiCo-P*x* (when *x* = 0.5, 1.0, 1.5, 2.0) cathode is enhanced. In addition, the high conductivity of BPL combined with the formation of the heterostructure optimizes its electronic structure and enhances electron transfer, enabling the electrode with a deep degree of reactivity and fast reaction kinetics, ultimately granting NiCo-P*x* (when *x* = 0.5, 1.0, 1.5, 2.0) excellent rate performance and lower resistance.

Further, the critical effect of CBPL is explored. For NiCo-P0.5 and NiCo-P1.0, as BPL increases, more high-conductivity active Ni_2_P/Co_2_P participate in the reaction and form a more extensive heterostructure with NiCo-LDH, resulting in more reactive sites, a faster electron transfer, and a deeper reaction depth of the electrode, representing a better performance of NiCo-P1.0. However, due to the more compact structure of nickel/cobalt phosphides compared with nickel/cobalt hydroxides, a further increase in the degree of phosphidation leads to a decrease in specific surface area and narrower ion transport channels, which significantly hinder ion adsorption and transport. This weakened ion transport limits the activation of the electrode material, as well as the rate and depth of electrochemical reactions, ultimately resulting in a reduction in the over amount of active material and sluggish reaction kinetics. Therefore, the weaker performance of NiCo-P1.5 compared with NiCo-P1.0 can be explained. When the phosphidizing degree is excessively high, the further reduction of the specific surface area, the further decrease in the overall active material, and the uneven distribution of active phosphidizing products caused by the emergence of phosphates ultimately lead to a decline in electrochemical performance. This is reflected in the inferior performance of NiCo-P2.0 compared with NiCo-P1.5. Thus, CBPL exhibits high conductivity and reactivity, and can synergistically interact with NiCo-LDH. Furthermore, CBPL optimally modulates the electronic structure, and surface activity of the nanohybrid cathode, achieving the optimal electron/ion transport rate and depth. This results in NiCo-P1.0 having the best material utilization and the fastest reaction kinetics, leading to the maximum energy supply as well as the best rate performance.

Subsequently, the reaction kinetics of NiCo-P1.0 is investigated. CV curves of NiCo-P1.0 at various scan rates are shown in Fig. 3e. According to the previous discussion, peaks 1 and 3 could be attributed to the electrode reaction of Ni(OH)_2_/Co(OH)_2_ while peaks 2 and 4 could mainly correspond to the electrode reaction of Ni_2_P/Co_2_P. Besides, there is no obvious change in the shape of CV curves when the scan rate increases from 1 to 5 mV s^−1^, indicating the excellent electrochemical reversibility and sufficient redox reaction of NiCo-P1.0. Based on the CV curves (Fig. 3e) and Eqs. S5 and S6 to describe ion storage behavior, the *b* values for the two pairs of redox peaks are firstly determined (Fig. 3f). Theoretically, the *b* value is 0.5 and 1.0 representing typical battery and capacitance behaviors correspondingly [60, 61]. It can be seen that the *b* values are 0.69 and 0.62 for the reaction of Ni(OH)_2_/Co(OH)_2_, while the b values are 0.73 and 0.87 for the reaction of Ni_2_P/Co_2_P. These results suggest that Ni(OH)_2_/Co(OH)_2_ and Ni_2_P/Co_2_P all experience diffusion/capacitive synergistically controlled storage processes, while the energy storage mechanism of Ni_2_P/Co_2_P tends to be a capacitive process. Further, the ratio of capacitive contribution and diffusion-controlled contribution can be further calculated by Eq. S7. For the peak 3 of NiCo-P1.0, the capacitive contributions are 38.20% at 1 mV s^−1^ and 58.02% at 5 mV s^−1^ (Fig. 3g). This suggests that NiCo-P1.0 exhibits battery-type behavior at low scan rates and capacitive-based kinetics behavior at high scan rates [5, 60, 61]. The increased capacitive contribution also leads to the excellent rate performance of NiCo-P1.0, which is attributed to the fast electron transfer, large specific surface area, and abundant active reaction sites provided by CBPL.

Density functional theory (DFT) is employed to further analyze the effect of CBPL on the electrode performance. Models of NiCo-LDH, NiCo-P1.0, and NiCo-P2.0 are constructed and optimized (Fig. 3h-j). The upper layer of the model represents the surface layer of the material, while the lower layer represents the inner material. Notably, for NiCo-P2.0, the presence of Ni_3_(PO_4_)_2_/Co_3_(PO_4_)_2_ in BPL is indicated in the upper layer. Based on the previous discussion, NiCo-LDH, NiCo-P1.0, and NiCo-P2.0 cathodes can be employed as working electrodes in the KOH alkaline solution. Consequently, the OH⁻ adsorption energies of NiCo-LDH, NiCo-P1.0, and NiCo-P2.0 are calculated to be − 0.77, − 1.31, and − 0.99 eV, respectively (Fig. 3k), indicating that NiCo-P1.0 has the most favorable OH^−^ adsorption for enhanced reaction kinetics. The difference in adsorption energies suggests that BPL promotes OH⁻ adsorption, while the presence of nickel phosphates and cobalt phosphates impedes OH⁻ adsorption, further confirming the superiority of CBPL.

In Fig. 3l–o, the total and partial density of states (DOS) of NiCo-LDH, NiCo-P1.0, and NiCo-P2.0 are compared. NiCo-P1.0 exhibits the highest charge density near the Fermi level, followed by NiCo-P2.0, with NiCo-LDH showing the lowest. This indicates that the electrical conductivity of NiCo-P1.0 is superior to that of NiCo-P2.0, and NiCo-P2.0 is more conductive than NiCo-LDH. Therefore, the superior conductivity of NiCo-P1.0 and NiCo-P2.0 compared with NiCo-LDH should be attributed to the high conductivity of BPL and the heterostructure formed between the BPL and NiCo-LDH. In addition, according to Fig. 3n, o, the DOS at the Fermi level of NiCo-P1.0 is mainly contributed by Ni and Co, while the DOS at the Fermi level of NiCo-P2.0 is mainly contributed by Ni and O. This indicates that the presence of nickel phosphates and cobalt phosphates reduces the electron mobility and conductivity of the electrode, confirming the superiority of CBPL. Furthermore, the p-states of O and P of NiCo-P1.0 overlap near the Fermi level, suggesting electron coupling between the upper and lower layers (Fig. S12). This synergistic behavior between CBPL and NiCo-LDH enhances electron transfer, resulting in excellent electrical conductivity. Therefore, the high conductivity of CBPL, the heterostructure formed between CBPL and NiCo-LDH, and the synergistic effect between CBPL and NiCo-LDH promote electron transfer, enabling NiCo-P1.0 to exhibit a high degree of reactivity and fast reaction kinetics.

**Fig. 4:**
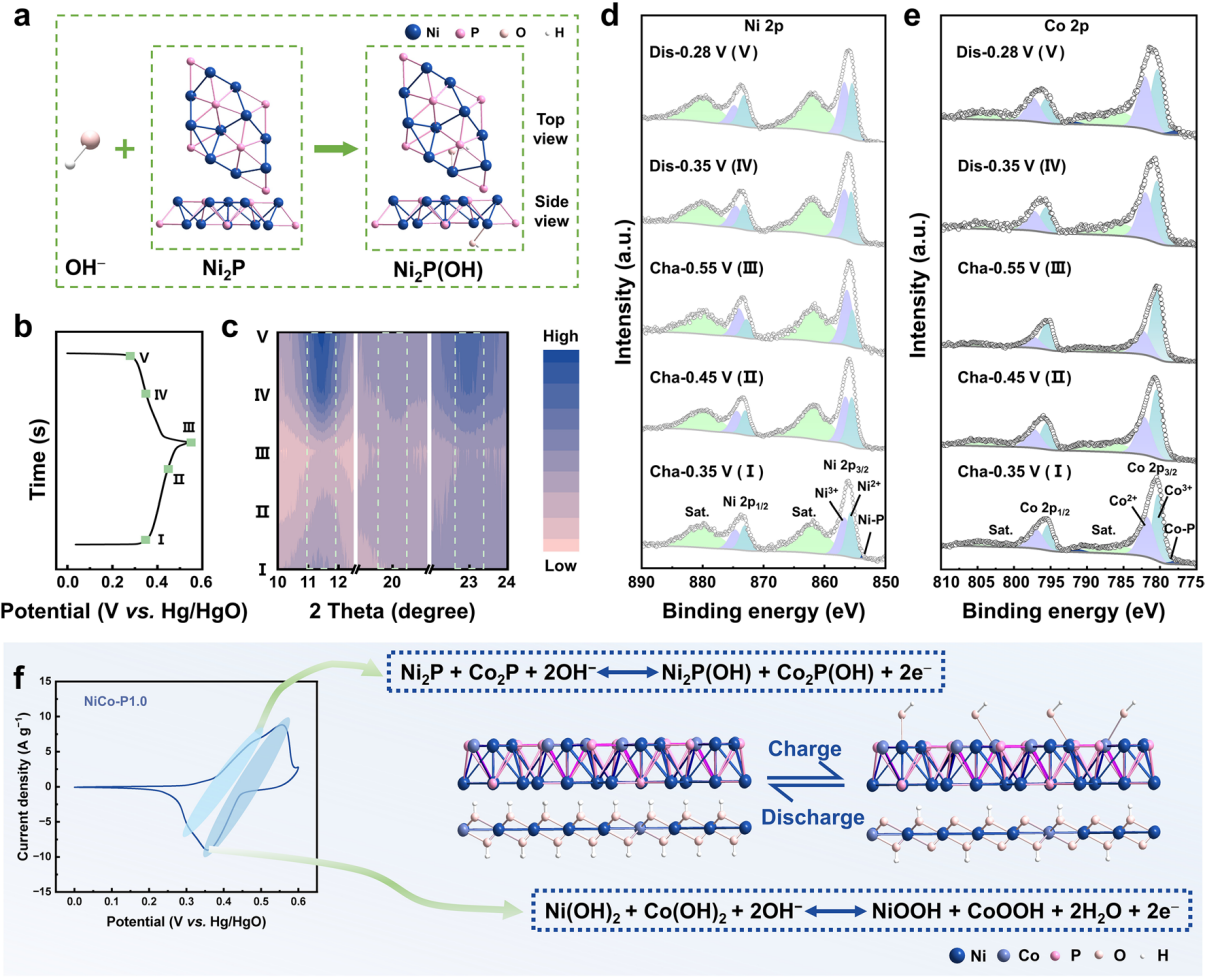
The energy storage mechanism of NiCo-P1.0 investigated by ex situ XRD, XPS, and DFT calculation. **a** Schematic of the formation of Ni_2_P(OH) by OH^−^ and Ni_2_P. **b** GCD curves and **c** ex situ XRD patterns collected during the whole charge–discharge process. High-resolution XPS spectra of **d** Ni 2*p* and **e** Co 2*p*, at various charged/discharged states. **f** Schematic diagram of the redox reactions corresponding to the redox peaks of NiCo-P1.0

Furthermore, the energy storage mechanism of NiCo-P1.0 is investigated as the cathode material for NZBs. According to the previous discussion, the NiCo-P1.0 electrode undergoes two electrode reactions. One reaction at high potential is the Ni(OH)_2_/Co(OH)_2_ electrode reaction, and the other at low potential is mainly the Ni_2_P/Co_2_P electrode reaction induced by CBPL. Generally, in alkaline electrolytes, the energy storage mechanism of Ni(OH)_2_/Co(OH)_2_ is considered to be the formation of NiOOH/CoOOH through reversible redox reactions [5, 11–13, 17, 62], while that of Ni_2_P/Co_2_P is considered to be the formation of (Ni_2_P)_*m*_(OH)_*n*_/(Co_2_P)_*m*_(OH)_*n*_ (m: n = 1: 1 or 1: 2) via reversible redox reactions [24, 25, 31, 49, 63]. Therefore, the models of Ni_2_P and Co_2_P are constructed and optimized (Fig. S13). Then, the models of (Ni_2_P)_*m*_(OH)_*n*_/(Co_2_P)_*m*_(OH)_*n*_ formed by OH⁻ binding to different sites on Ni_2_P/Co_2_P are presented (Figs. S14–S17). By comparing the  binding energy (Tables S4 and S5), Ni_2_P(OH)/Co_2_P(OH) has lower energy than Ni_2_P(OH)_2_/Co_2_P(OH)_2_, proving that the former is theoretically more stable. Then, the most stable models for Ni_2_P(OH)/Co_2_P(OH) are identified (Fig. S18). Therefore, as shown in Figs. 4a and S19, the electrode reaction of CBPL is likely to be Eq. 2.

In order to further investigate the structural evolution and energy storage mechanism of NiCo-P1.0 during the charging-discharging cycle, five different states of charge are selected (Fig. 4b). The ex situ XRD is carried out to understand the crystal structure evolution (Figs. 4c and S20). It can be observed that during the charge–discharge process, the peaks around 11.4°, 20.1°, and 22.7° show significant changes. The peak around 20.1° is attributed to hexagonal Co(OH)_2_ (PDF#001–0357), while the other two peaks are attributed to hexagonal Ni(OH)_2_ (PDF#038–0715). Therefore, the content of Ni^2+^/Co^2+^ continues to decrease during charging and increase during discharging, corresponding to the redox reactions of Ni(OH)_2_/Co(OH)_2_. Notably, during the low-voltage charging process (from stage I to II), the Ni^2+^/Co^2+^ content decreases slightly, while in the high-voltage charging process (from stage II to III), it decreases significantly. During the high-voltage discharging process (from stage III to IV), the Ni^2+^/Co^2+^ content increases significantly, while during the low-voltage discharging process (from stage IV to V), it increases slightly. Thus, the redox reactions of Ni(OH)_2_/Co(OH)_2_ occur throughout the charge–discharge process, mainly in the high-voltage region, corresponding to peaks 1 and 3 in the CV curves (Fig. 3e).

Furthermore, the surface chemical composition and valence states of NiCo-P1.0 during the charge–discharge process are evaluated using XPS. For the five charge–discharge states (from stage I to V), the XPS survey spectra show the presence of Ni, Co, O, and P elements (Fig. S21a). It is noteworthy that the signals related to the P element are very weak at higher voltage charge–discharge states (II-IV) (Figs. 4d, e and S21b), which is attributed to surface oxidation [42, 52, 55, 58]. In the high-resolution Ni 2*p* XPS spectra (Fig. 4d), distinct binding energy peaks corresponding to Ni^2+^, Ni^3+^, and Ni–P can be observed [9, 41, 42, 49]. Similarly, obvious binding energy peaks corresponding to Co^2+^, Co^3+^, and Co-P can be observed in the high-resolution Co 2*p* XPS spectra (Fig. 4e) [9, 41, 49]. Notably, the XPS results indicate that Ni^3+^/Co^3+^ consistently increases during the charging process and decreases during discharging. Specifically, during the low-voltage charging process (from stage I to II), Ni^3+^/Co^3+^ exhibits a slight increase, whereas the high-voltage charging process (from stage II to III) results in a significant increase. Conversely, during the high-voltage discharging process (from stage III to IV), Ni^3+^/Co^3+^ shows a marked decrease, while the low-voltage discharging process (from stage IV to V) leads to a slight decrease. This is consistent with the XRD patterns, revealing that the redox reactions of Ni(OH)_2_/Co(OH)_2_ occur throughout the charge–discharge process, predominantly in the high-voltage region, corresponding to peaks 1 and 3 in the CV curves (Fig. 3e). Therefore, the energy storage mechanism of NiCo-P1.0 in the alkaline electrolyte is still dominated by the redox reactions of Ni(OH)_2_/Co(OH)_2_, which tend to occur at higher voltage. Furthermore, redox reactions of Ni_2_P/Co_2_P also contribute, which is at a relatively low voltage.

**Fig. 5:**
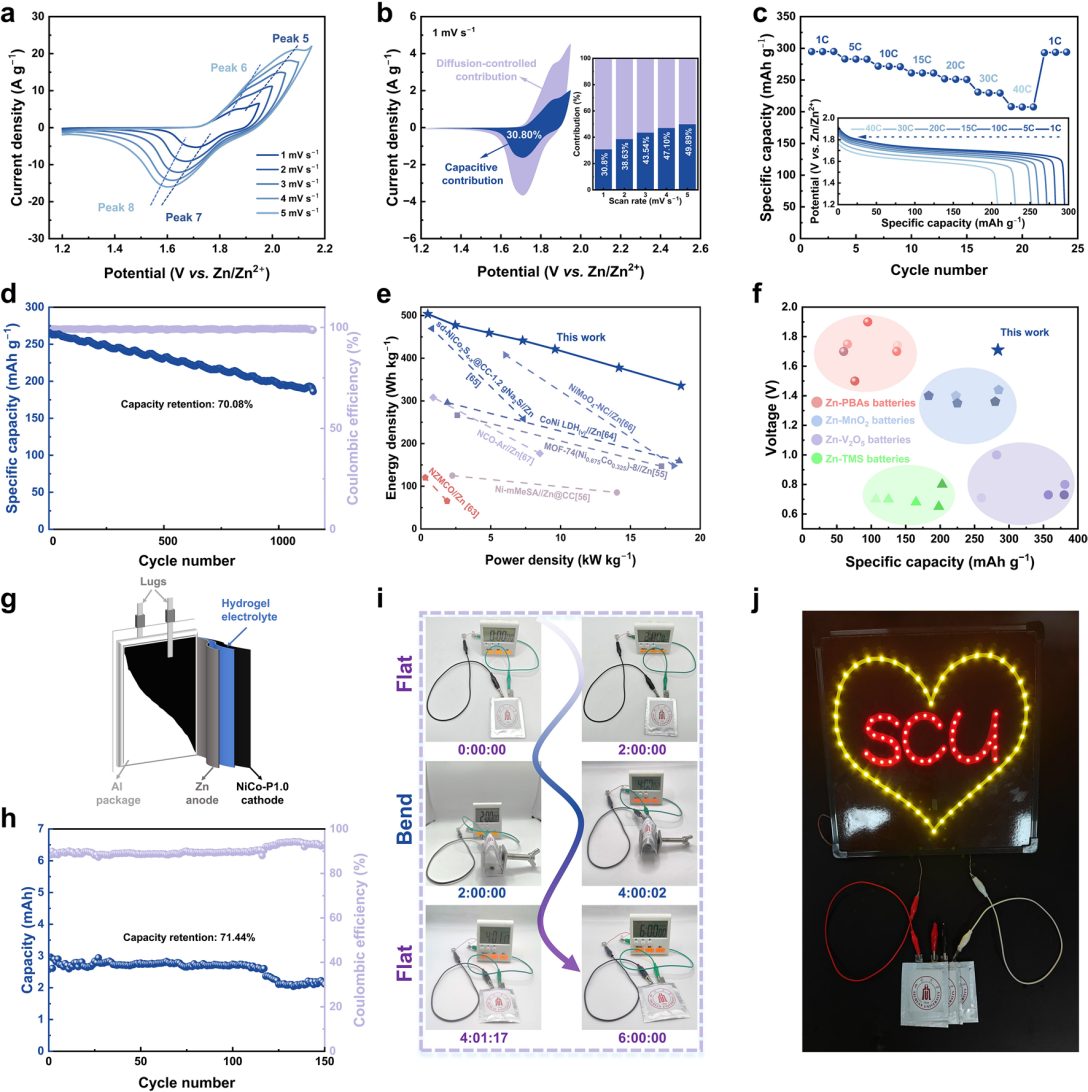
Electrochemical performances of the NiCo-P1.0//Zn battery. **a** CV curves at different scan rates. **b** Diffusion-controlled and capacitive contributions at 1 mV s^−1^ (insert: the calculated capacitive contribution at different scan rates). **c** Rate performance at different current densities from 1 to 40C (insert: the discharge curves at different current densities). **d** Long-time cycling performance at 5C. **e** Ragone plots of the NiCo-P1.0//Zn battery and other NZBs. **f** Ragone plots of average output voltage *vs.* specific capacity for NiCo-P1.0 and some representative cathodes of aqueous ZBs (PBAs: Prussian blue analogues; TMSs: transition metal sulfides). Electrochemical performances of the flexible quasi-solid-state pouch cell. **g** Assembly diagram of the flexible pouch cell. **h** Cycling performance at 1C. **i** Digital photographs showing an electronic timer driven by a pouch cell at a flat-bend-flat deformation state for 6 h. **j** Digital photograph of three pouch batteries connected in series to power a neon sign

To further investigate the practical applications of NiCo-P1.0, the NiCo-P1.0//Zn battery is assembled using NiCo-P1.0 as the cathode, 3 M KOH with saturated ZnO solution as the electrolyte, and Zn obtained by electrodeposition as the anode. The CV curves of the NiCo-P1.0//Zn battery are shown in Fig. 5a, where the redox peaks shift slightly when the scan rate increases from 1 to 5 mV s^−1^, indicating the potential for excellent rate performance. Notably, two distinct pairs of redox peaks can be observed, indicating the reaction of Ni(OH)_2_/Co(OH)_2_ and Ni_2_P/Co_2_P. Therefore, the overall electrochemical reaction in the NiCo-P1.0//Zn battery can be described as Eq. 4. Furthermore, the ratio of capacitive contribution and diffusion-controlled contribution was calculated by Eq. S7. For peak 7, the capacitive contribution is 30.80% at 1 mV s^−1^ and 49.89% at 5 mV s^−1^ (Fig. 5b). The increased capacitive contribution also suggests the excellent rate performance of NiCo-P1.0//Zn battery, attributed to the NiCo-P1.0 cathode.

According to Eq. S2 and Fig. 5c, the average discharge capacities of NiCo-P1.0//Zn are 294.80, 282.79, 271.44, 260.94, 251.01, 229.44, and 207.12 mAh g^−1^ from 1 to 40C, with an impressive capacity retention of 70.26% at 40C. Moreover, when the current density is restored to the initial 1C, the discharge capacity remains at 293.63 mAh g^−1^, almost unchanged from the initial value. Besides, the discharge curves of the NiCo-P1.0//Zn battery from 1 to 40C are shown in Fig. 5c (insert). It is shown that there is still an obvious discharge plateau even when the current density is expanded to 40C. Therefore, the NiCo-P1.0//Zn battery demonstrates excellent rate performance, attributed to the outstanding properties of the NiCo-P1.0 cathode. Figure S22 demonstrates the high electrical conductivity of the NiCo-P1.0//Zn battery, attributed to the excellent electronic conductivity and ion transport of the NiCo-P1.0 cathode.

Metal hydroxides as the cathode often suffer from lower cycling performance due to incomplete oxidation during the charging process [64, 65]. Therefore, when testing the cycling life of the NiCo-P1.0//Zn battery, besides the conventional GCD method (Fig. S23), a short period of constant voltage charging after constant current charging at 5C is used (Fig. S24). As observed in Fig. 5d, a high cycling performance is achieved with a capacity retention of 70.08% over 1150 cycles with Coulombic efficiency nearly 100%. Furthermore, the morphology and structure of NiCo-P1.0 after cycling were characterized to analyze its capacity decay mechanism. During cycling, nickel-based cathodes typically undergo phase transitions, and long-term cycling may lead to the formation of irreversible phases, resulting in capacity degradation [17, 25, 54, 66–70]. After cycling, the nanosheet structure of NiCo-P1.0 becomes much more evident (Fig. S25), indicating structural reconstruction. XRD pattern (Fig. S26) demonstrates notable changes, particularly the intensity variation of the characteristic peak at 25.92° corresponding to the (002) crystal plane of C (PDF#00–058-1638), which primarily resulted from the unavoidable incorporation of carbon fibers during sample preparation when scraping samples from the carbon cloth substrate. More importantly, the diffraction peaks associated with Ni/Co phases exhibit substantially reduced intensity, suggesting significant depletion of Ni/Co species in the cycled cathode. Consistent with the previous discussion and studies [12, 22], this indicates the electrochemical transformation of Ni/Co components into Ni(OH)_2_/Co(OH)_2_ phases during cycling. Figure S27 reveals that the characteristic diffraction peaks shift to a lower angle, suggesting an increase in interlayer spacing. Since γ-NiOOH exhibits a larger interplanar spacing than those of β-NiOOH and Ni(OH)_2_ phases, this peak shift may indicate the formation of γ-NiOOH. Also, it is possible that the interlayer expansion arises from structural strain relaxation or other phase transitions [66–70]. Therefore, the capacity decay of NiCo-P1.0 after cycling may be primarily attributed to the irreversible crystal phase transition, which reduces the electrochemical reversibility, as well as structural degradation, including interlayer expansion and material pulverization [66–70]. These changes increase internal resistance and accelerate the loss of active material, ultimately compromising its electrochemical performance.

The energy and power densities of the NiCo-P1.0//Zn battery based on the active mass loading of the cathode are calculated according to Eqs. S3 and S4 and compared with those of other reported NZBs using a Ragone plot (Fig. 5e). The NiCo-P1.0//Zn battery achieved a maximum power density of 18.62 kW kg^−1^ (at an energy density of 335.57 Wh kg^−1^) and a maximum energy density of 503.62 Wh kg^−1^ (at a power density of 493.55 W kg^−1^), which is significantly better than those reported NZBs in Table S6 [56, 57, 62, 71–74]. Furthermore, the electrochemical performance of NiCo-P1.0//Zn is compared with other representative aqueous batteries. Both high voltage and high capacity are crucial criteria for evaluating the performance and practicability of batteries. The Ragone plot (Fig. 5f) further described a high specific capacity of 294.80 mAh g^−1^ with a high working voltage of 1.71 V of NiCo-P1.0//Zn battery, superior to the reported results in some representative publications [75–96]. More detailed information is summarized in Table S7.

Subsequently, to further verify the practicability and expandability of NiCo-P1.0, a quasi-solid-state pouch cell is assembled with NiCo-P1.0 as the cathode, hydrogel as the electrolyte, and zinc foil as the anode (Fig. 5g). A pouch battery with a size of 2 cm $\times$ 2 cm exhibits stable cycling over 150 cycles at 1C with a capacity retention of 71.44% (Fig. 5h). The GCD curves are shown in Fig. S28. It is worth noting that the pouch cell exhibits flexibility. As shown in Fig. 5i, the pouch cell can continuously and stably power a timer for 6 h under the deformation states of flat-bent-flat. In addition, three pouch cells connected in series can stably and continuously powder a neon sign consisting of 34 red LEDs and 46 yellow LEDs (Fig. 5j). Above all, it can be inferred that NiCo-P1.0, with excellent electrochemical performance, holds great promise for applications in ZBs.

## Conclusion

In summary, surface modification with CBPL can effectively leverage its high conductivity and the construction of heterostructures to enhance electronic conductivity and improve reaction kinetics. Additionally, the CBPL can serve as the active site for OH^−^ adsorption and synergistically interact with LDH, effectively enhancing the energy supply. The nanohybrid cathode with CBPL (NiCo-P1.0) demonstrates a high capacity of 286.64 mAh g^−1^ at 1C and the superb rate performance (a capacity retention of 72.22% at 40C). The assembled NiCo-P1.0//Zn battery achieves excellent energy/power density (503.62 Wh kg^−1^/18.62 kW kg^−1^). In addition, the utility of NiCo-P1.0 is also proved in the flexible quasi-solid-state soft pack cells. This work provides an effective and scalable strategy for composite design through surface modification with bifunctional metal phosphide layers, paving the way for high-performance cathodes with highly promising applications in aqueous energy storage.

## Supplementary Information

Below is the link to the electronic supplementary material.

Supplementary file1 (DOCX 6528 KB)
