# Supplementary material for: Critical Bimetallic Phosphide Layer Enables Fast Electron Transfer and Extra Energy Supply for Flexible Quasi-Solid-State Zinc Batteries
Source: Nanomicro Lett. 2025 May 21;17:266. doi: 10.1007/s40820-025-01784-3 (PMC12095782; doi:10.1007/s40820-025-01784-3)
Supplement: Supplementary file 1 — Supplementary file1 (DOCX 6528 KB) [file 40820_2025_1784_MOESM1_ESM.docx]

Supporting Information for

**Critical Bimetallic Phosphide Layer Enables Fast Electron Transfer and Extra Energy Supply for Flexible Quasi-Solid-State Zinc Batteries**

Leixin Wu^1, †^, Linfeng Lv^1, 2, †^, Yibo Xiong^1^, Wenwu Wang^1^, Xiaoqiao Liao^1^, Xiyao Huang^1^, Ruiqi Song^1^, Zhe Zhu^3^, Yixue Duan^1^, Lei Wang^1^, Zeyu Ma^1^, Jiangwang Wang^1^, Fazal ul Nisa^1^, Kai Yang^1^, Muhammad Tahir^4^, Longbing Qu^5^, Wenlong Cai^6^, and Liang He^1, 7, 8,^ *

^1^ School of Mechanical Engineering, State Key Laboratory of Intelligent Construction and Healthy Operation and Maintenance of Deep Underground Engineering, Sichuan University, Chengdu, 610065, People’s Republic of China

^2^ Department of Mechanical Engineering, City University of Hong Kong, Tat Chee Avenue, Hong Kong, 999077, People’s Republic of China

^3^ School of Chemistry, Faculty of Science, University of New South Wales, Sydney, NSW, 2052, Australia

^4^ School of Mechatronical Engineering, Beijing Institute of Technology, Beijing, 100081, People’s Republic of China

^5^ Department of Chemical Engineering, The University of Melbourne, Melbourne, VIC, 3010, Australia

^6^ College of Materials Science and Engineering, Sichuan University, Chengdu, 610064, People’s Republic of China

^7^ Med+X Center for Manufacturing, West China Hospital, Sichuan University, Chengdu, 610041, People’s Republic of China

^8^ Yibin Industrial Technology Research Institute of Sichuan University, Yibin R&D Park of Sichuan University, Yibin, 644005, People’s Republic of China

^†^ Leixin Wu and Linfeng Lv have contributed equally to this work.

*Corresponding author. E-mail: [hel20@scu.edu.cn](mailto:hel20@scu.edu.cn) (Liang He)

**S1 Experimental Section**

**S1.1 Materials characterization**

The morphologies of the samples were observed by scanning electron microscope (SEM, Zeiss Gemini 300). The nanostructures of the electrode were observed by transmission electron microscope (TEM, FEI Talos F200x). X-ray diffraction (XRD) data were obtained using a Rigaku MiniFlex 600 diffractometer with Cu-Kα radiation (λ = 1.5406 Å) under 40 kV and 30 mA, at a scan rate of 1° min^‒1^. XPS results were recorded via a Thermo Scientific K-Alpha electron spectrometer using Al Kα irradiation ($hv$ = 1486.6 eV). The specific surface area values were calculated using the Brunauer-Emmett-Teller (BET) method on a Micromeritics ASAP 2460.

**S1.2 Electrochemical measurements**

Electrochemical measurements of Ni(OH)_2,_ NiCo-LDH, NiCo-P0, NiCo-P0.5, NiCo-P1.0, NiCo-P1.5, and NiCo-P2.0 cathodes were carried out with a three-electrode system in 3 M KOH aqueous electrolyte. A Pt plate was used as the counter electrode and a Hg/HgO electrode was the reference electrode. Before all electrochemical measurements, all of the cathodes were activated after 100 cyclic voltammetry (CV) cycles under 0-0.9 V at 10 mV s^−1^. Electrochemical measurements of NiCo-P1.0//Zn battery were performed in a two-electrode system with 3 M KOH solution and saturated ZnO as the electrolyte. For the pouch cells, Ti foil was used as the cathode tab, NiCo-P1.0 as the cathode (mass loading: 5 mg cm^−2^), hydrogel as the electrolyte, and zinc foil as the anode. CV and electrochemical impedance spectroscopy (EIS) tests were performed on the MULTI AUTOLAB M204 and CHI660E electrochemical workstations. EIS tests were conducted using a sinusoidal perturbation signal of 5 mV in the frequency range from 100 kHz to 10 mHz. Galvanostatic charge/discharge (GCD) curves and cycling performance were measured at a potential range using the battery test system (CT-3002A, Wuhan Land, Wuhan, People’s Republic of China) under the ambient environment.

**S1.3 Calculations**

The specific capacity *C* (mAh g^−1^) of the working electrodes in the three-electrode system was calculated from discharge curves according to the following equation (S1).

$C=\frac{I\times\Delta t}{m}$ (S1)

Where *I* (mA) is the discharging current, ∆*t* (h) is the discharging time, and *m* (g) is the active mass loading of the electrodes (2 mg).

Alternatively, the specific capacity of the NZBs (*C*_battery_) in the two-electrode system was calculated from the discharging curves according to the following equation (S2).

$C_{battery}=\frac{I\times\Delta t}{m}$ (S2)

Where *I* (mA) is the discharging current, ∆*t* (h) is the discharging time, and *m* (g) is the active mass loading of the cathode (2 mg).

The energy density (*E*) and power density (*P*) of the battery were calculated from the following equations (S3-S4).

$E=\frac{I\int_{0}^{\Delta t} U(t)dt}{m}$ (S3)

$P=\frac{E}{\Delta t}$ (S4)

Where *E* (Wh kg^−1^) is the energy density, *I* (mA) is the discharging current, *U*(t) is the voltage during discharge (V), d*t* is the time differential, ∆*t* (h) is the discharging time, and *m* (g) is the active mass loading of the cathode (2 mg). *P* (kW kg^−1^) is the power density, and ∆*t* (h) is the discharging time.

The kinetic of capacitive contribution can be obtained according to the CV curves at various scan rates. The relationship between current ($i$) and scan rate ($v$) can be expressed as:

$i=av^{b}$ (S5)

$\log i=\log a+b\log v$ (S6)

where *a* and *b* are constant which can be obtained from log(*v*) versus log(*i*) plots.

Furthermore, the ratio of capacitive contribution could be described as the following equation (S7).

$i(v)=k_{1}v+k_{2}v^{1/2}$ (S7)

Where $k_{1}v$ and $k_{2}v^{1/2}$ correspond to the capacitive and diffusion-controlled contributions, respectively.

**S2 Computational Methods**

Density functional theory (DFT) calculations were performed using Materials Studio to investigate the density of states (DOS) of NiCo-LDH, NiCo-P1.0, and NiCo-P2.0, as well as the adsorption energy of hydroxide on the material’s surfaces.

Geometry optimizations were carried out within the forcite module under convergence thresholds of 2.0 $\times$ 10^−5^ kcal mol^−1^ for energy and 1.0 $\times$ 10^−3^ kcal mol^−1^ Å^−1^ for force. The universal force field was employed for van der Waals interactions, while electrostatic interactions were handled using partial charges derived from the QEq method with the Ewald summation.

DOS was computed using the CASTEP module with the Perdew-Burke-Ernzerhof (PBE) functional under the generalized gradient approximation (GGA). The calculations adopted an energy cutoff of 310.00 eV and a Monkhorst-Pack k-point grid of (3 $\times$ 3 $\times$ 3). A self-consistent field (SCF) tolerance of 2.0 $\times$ 10^−6^ was set to ensure convergence.

Adsorption energy was calculated using the DMol³ module with the GGA-PBE and DND basis sets. Grimme’s dispersion corrections were included, and spin-unrestricted calculations were performed on a Monkhorst-Pack grid with a spacing of 0.08 Å^−1^. A SCF tolerance of 1.0 $\times$ 10^−5^ was set to ensure the reliability of the results.

Adsorption energy Δ*E*_adsorption_ was determined as equation (S8).

∆*E*_adsorption_ = *E*_adsorbate-adsorbent_ − (*E*_adsorbate_ + *E*_adsorbent_) (S8)

Where *E*_adsorbate-adsorbent_ is the total energy of the system, *E*_adsorbate_ is the energy of the isolated adsorbate (hydroxide), and *E*_adsorbent_ is the energy of the isolated adsorbent (NiCo-LDH, NiCo-P1.0, and NiCo-P2.0).

**S3 Supplementary Figures and Tables**


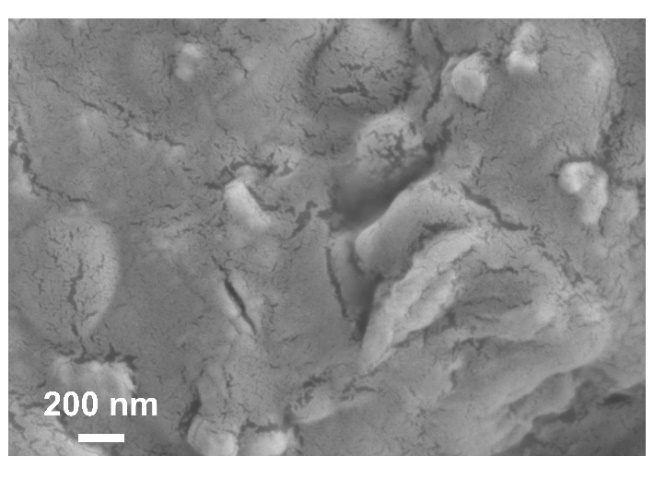


**Fig. S1** SEM image of NiCo-P3.0


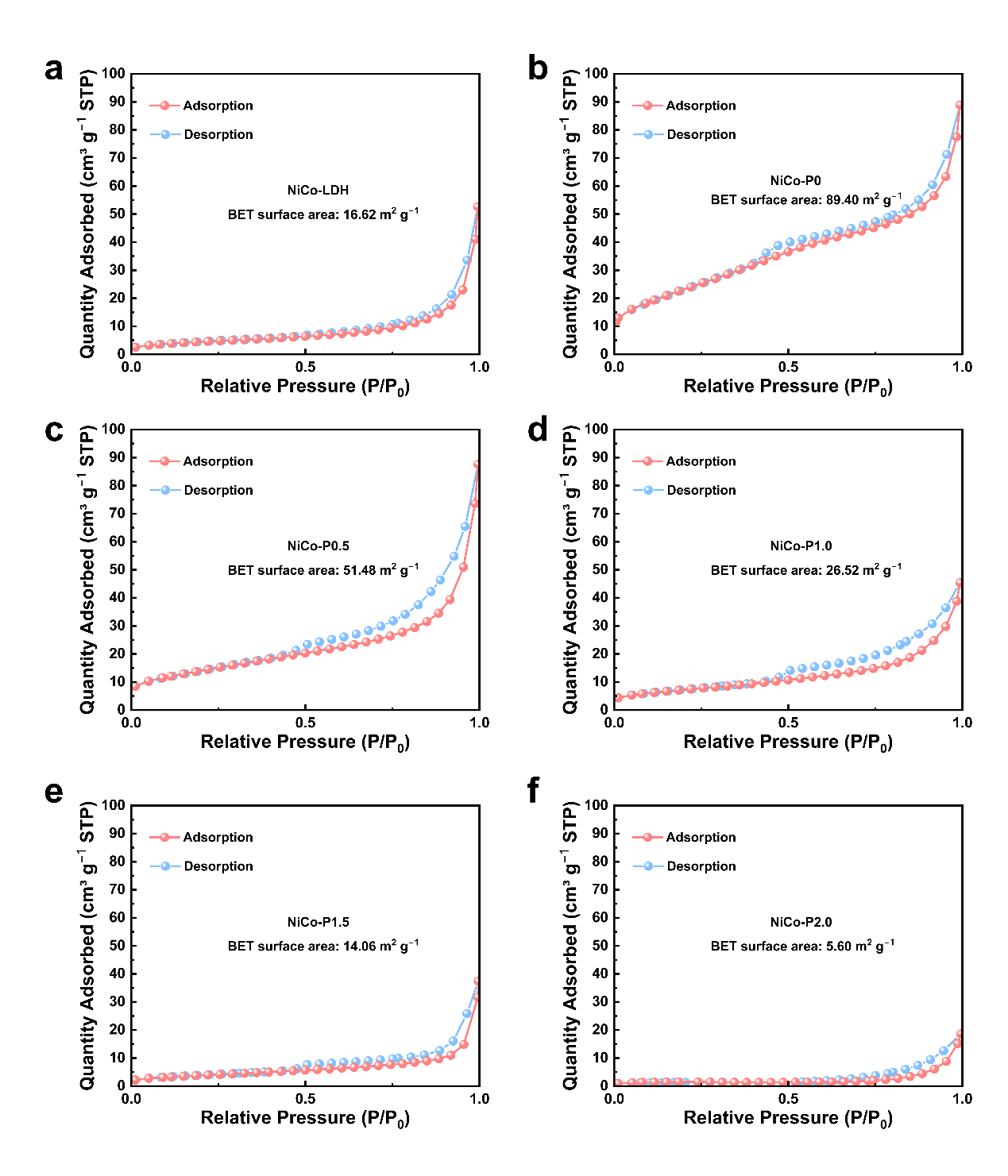


**Fig. S2** N_2_ adsorption-desorption isotherms of **a** NiCo-LDH, **b** NiCo-P0, **c** NiCo-P0.5, **d** NiCo-P1.0, **e** NiCo-P1.5, and **f** NiCo-P2.0


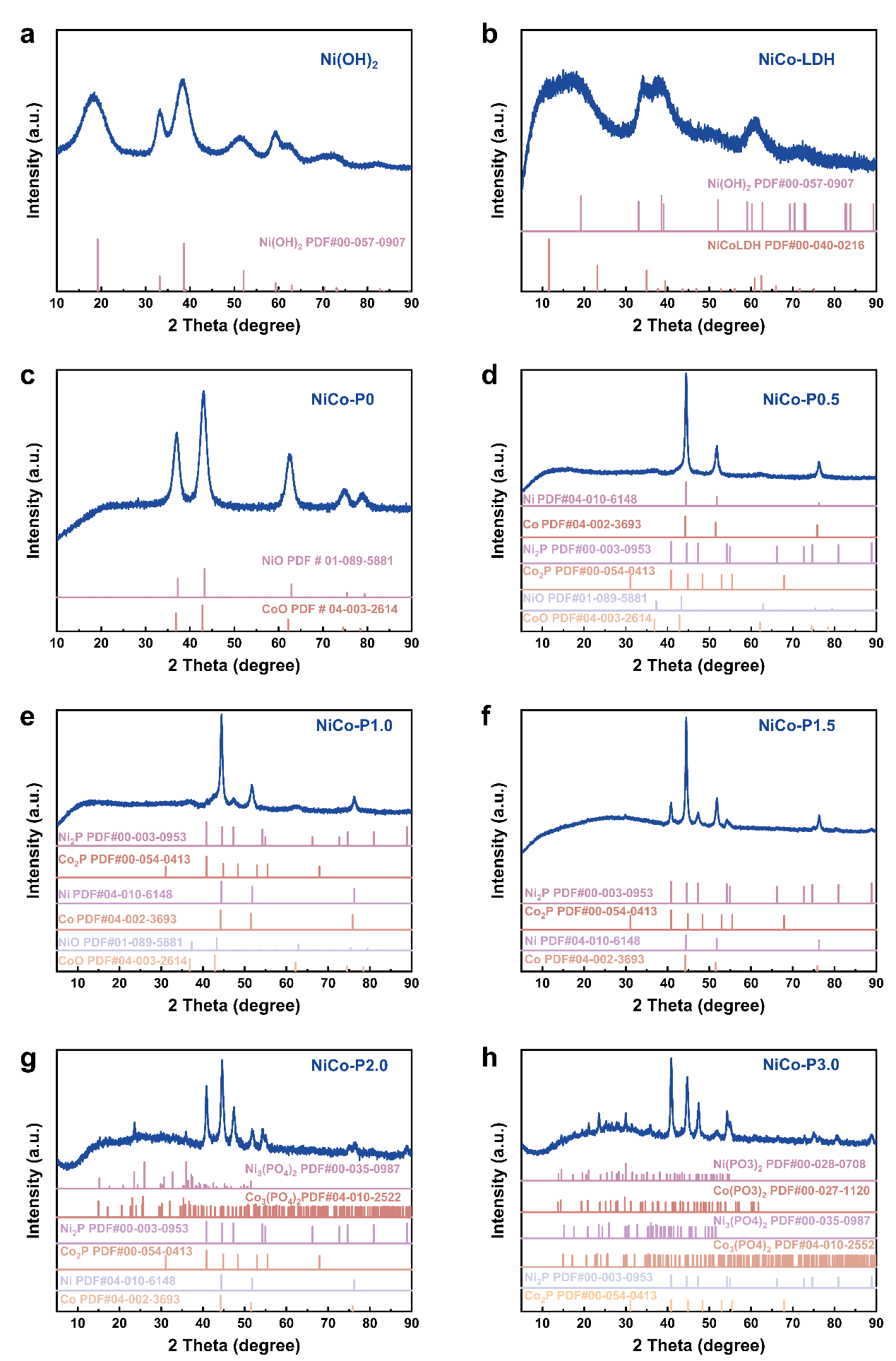


**Fig. S3** XRD patterns of **a** Ni(OH)_2_, **b** NiCoLDH, **c** NiCo-P0, **d** NiCo-P0.5, **e** NiCo-P1.0, **f** NiCo-P1.5, **g** NiCo-P2.0, and **h** NiCo-P3.0


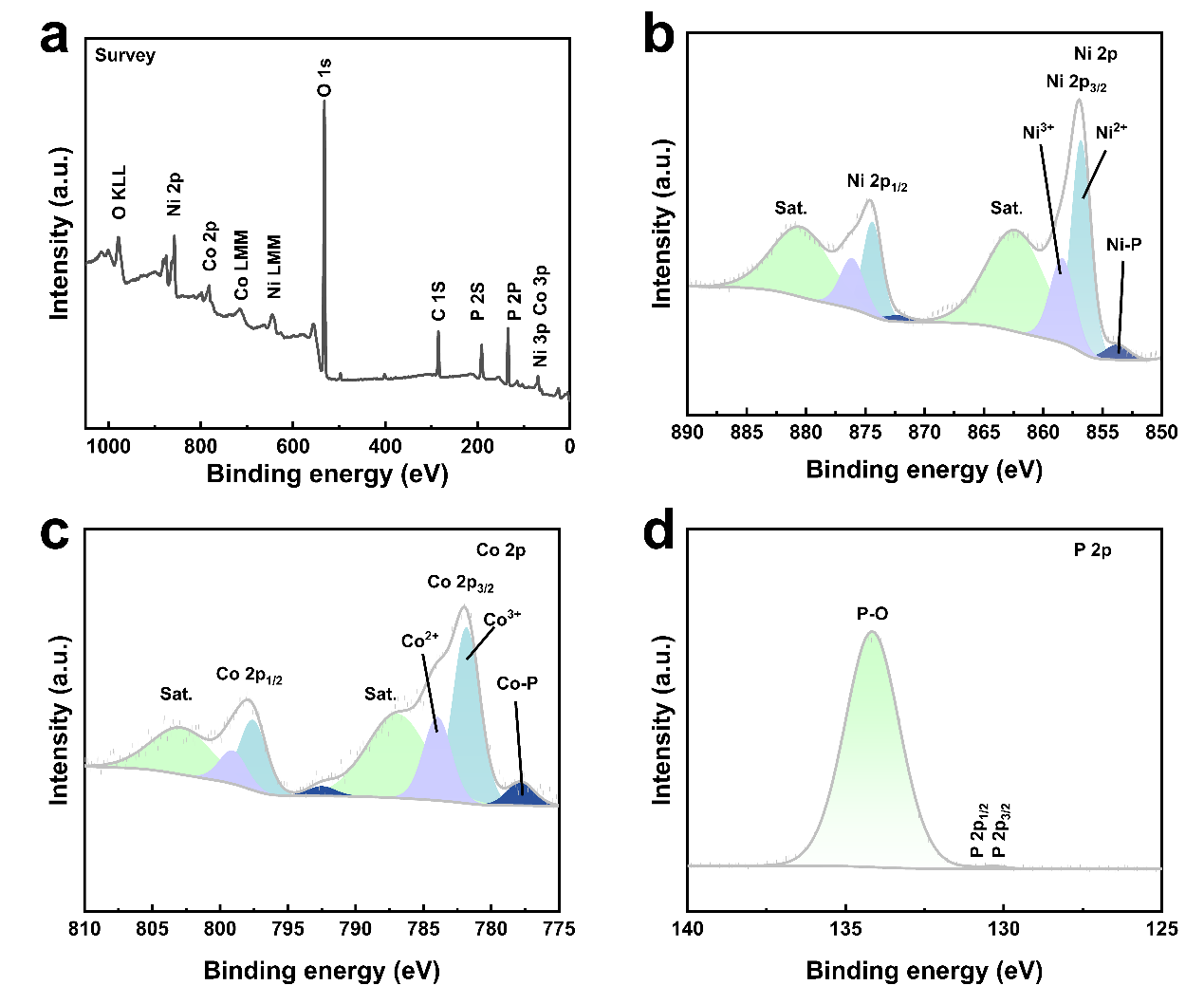


**Fig. S4 a** XPS survey spectrum and high-resolution **b** Ni *2p*, **c** Co *2p*, and **d** P 2p XPS spectra of NiCo-P3.0


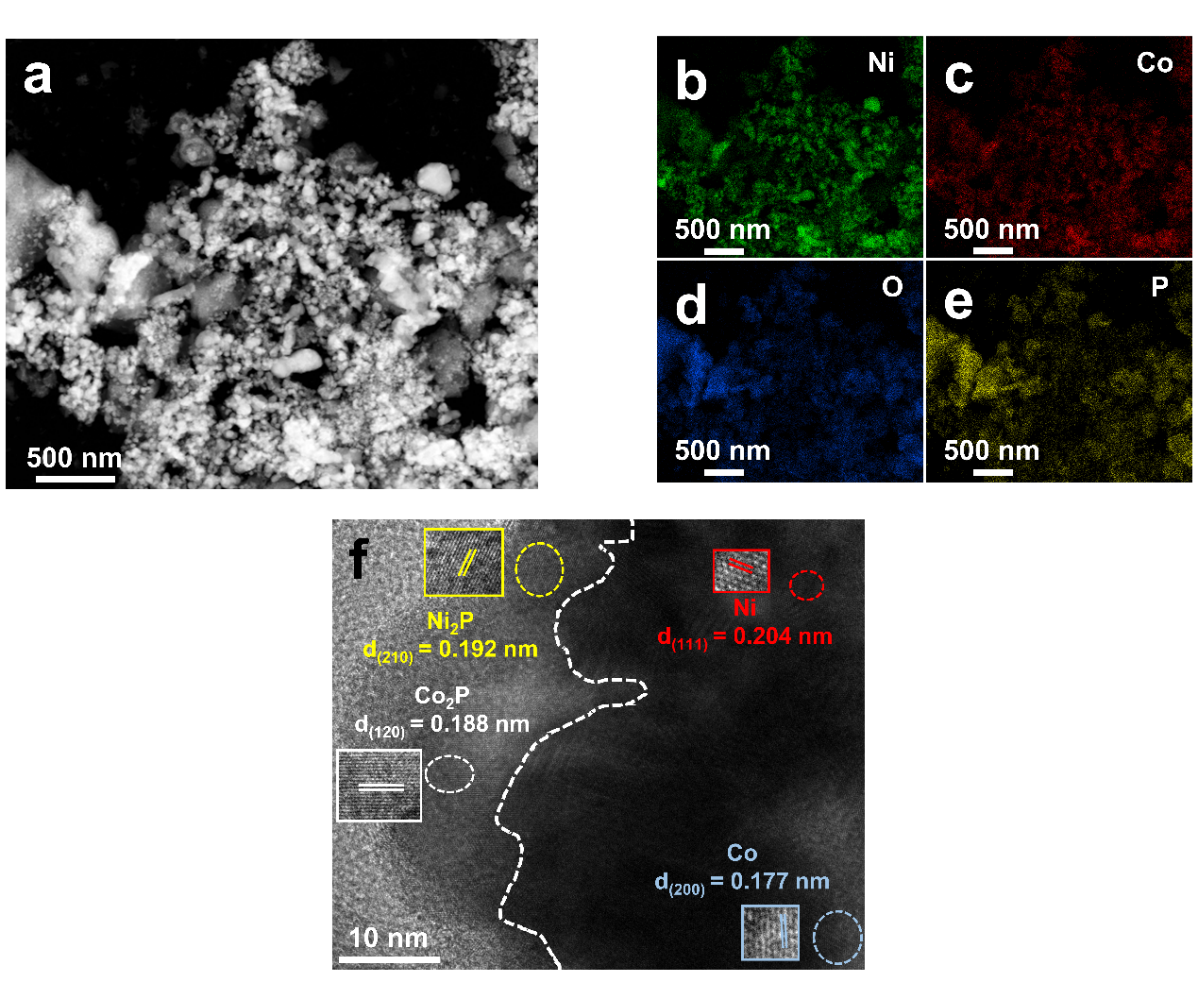


**Fig. S5** **a** TEM image, **b-e** elemental mapping images, and **f** HRTEM image of NiCo-P1.0


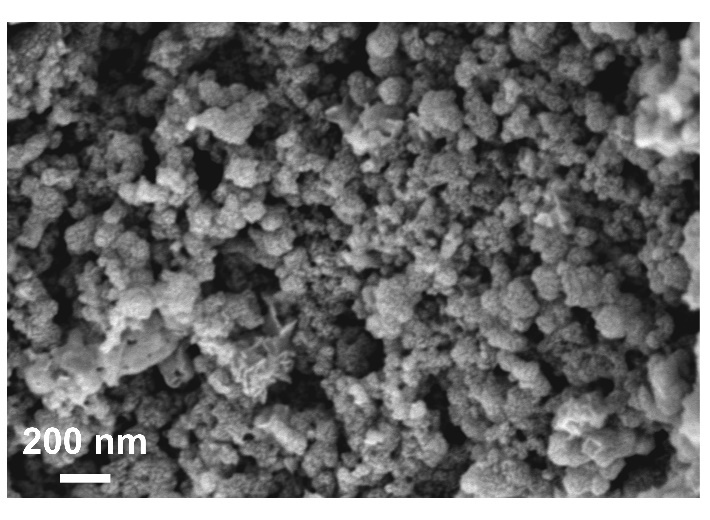


**Fig. S6** SEM image of NiCo-P1.0 after EA


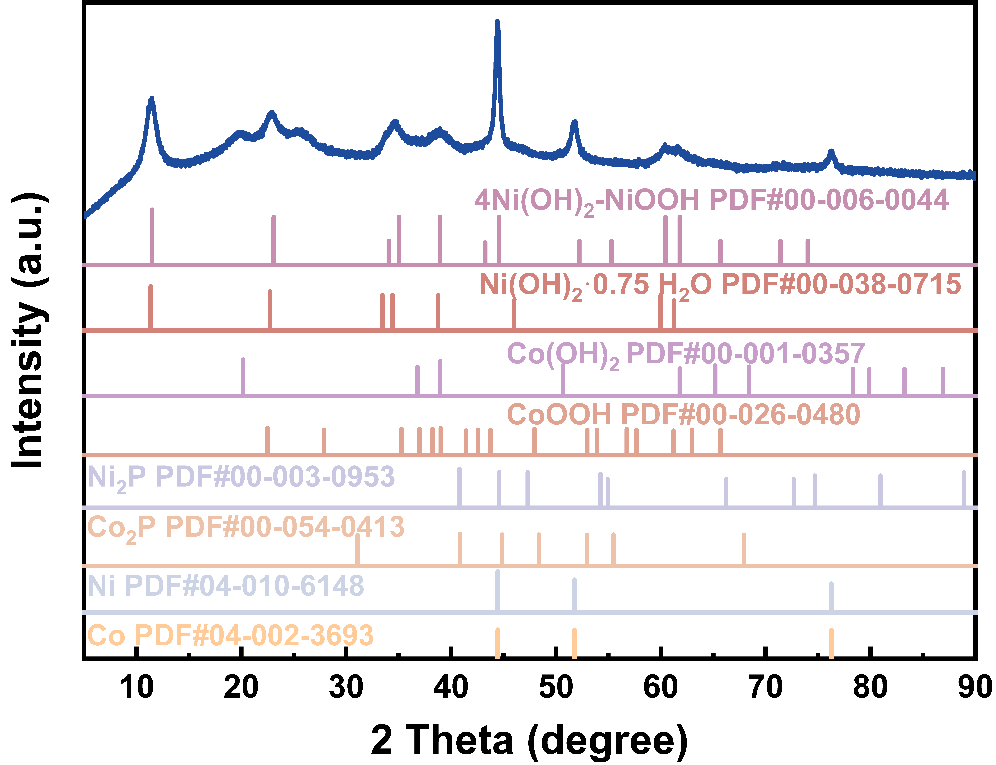


**Fig. S7** XRD pattern of NiCo-P1.0 after EA


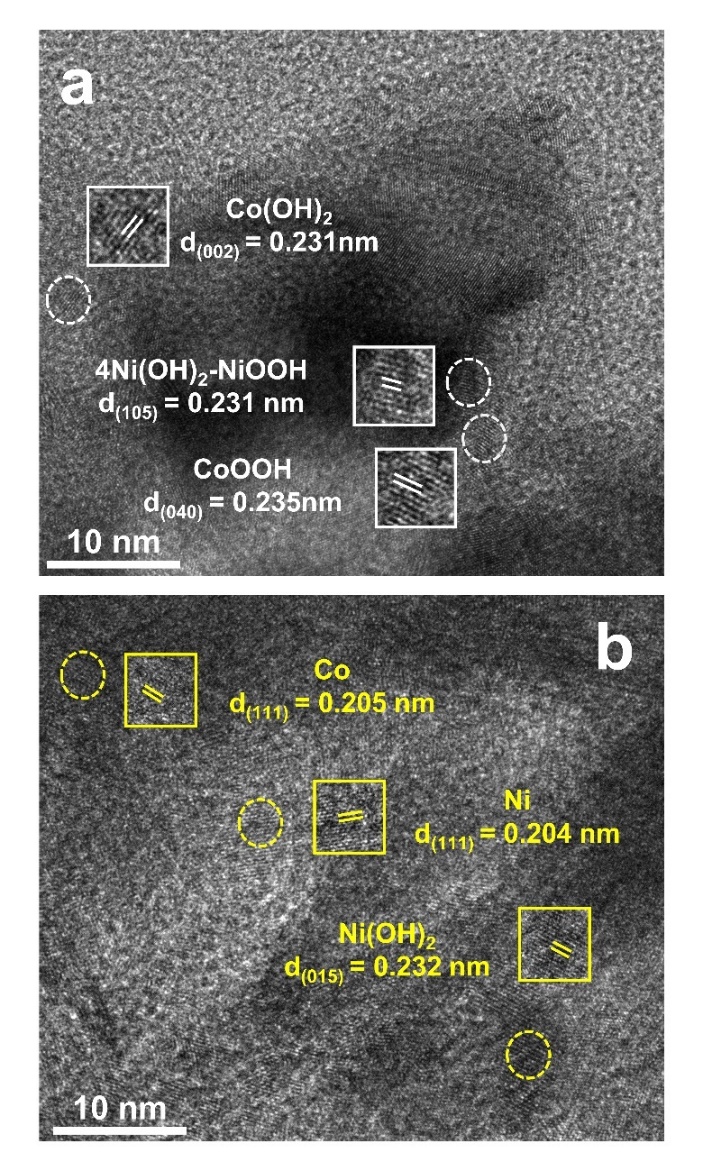

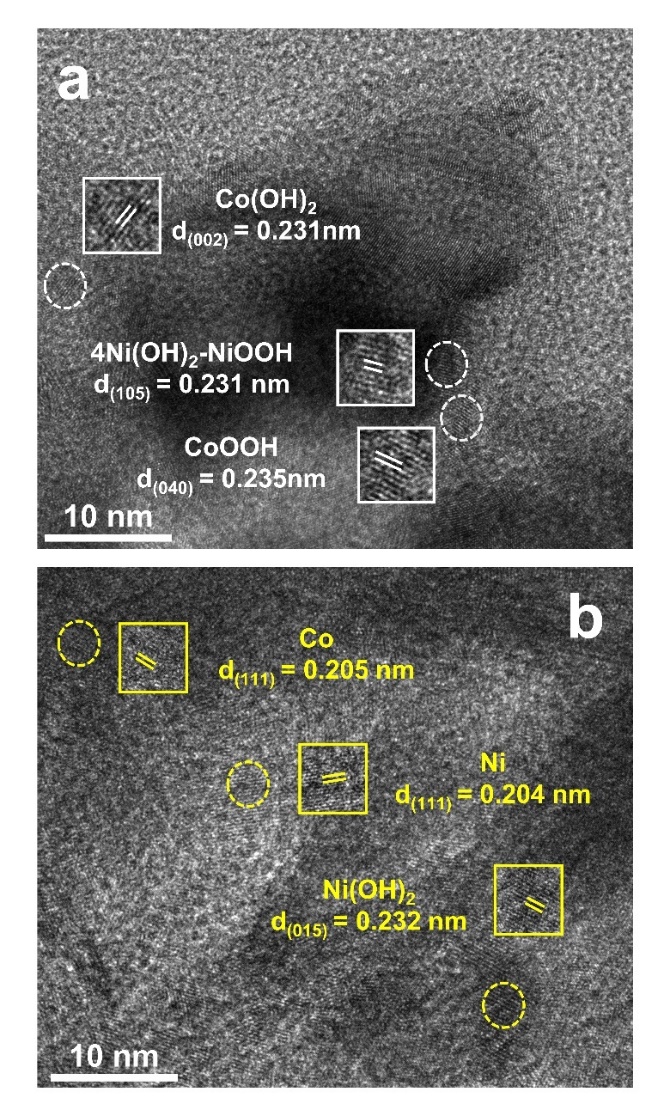


**Fig. S8** HRTEM images of NiCo-P1.0 after EA


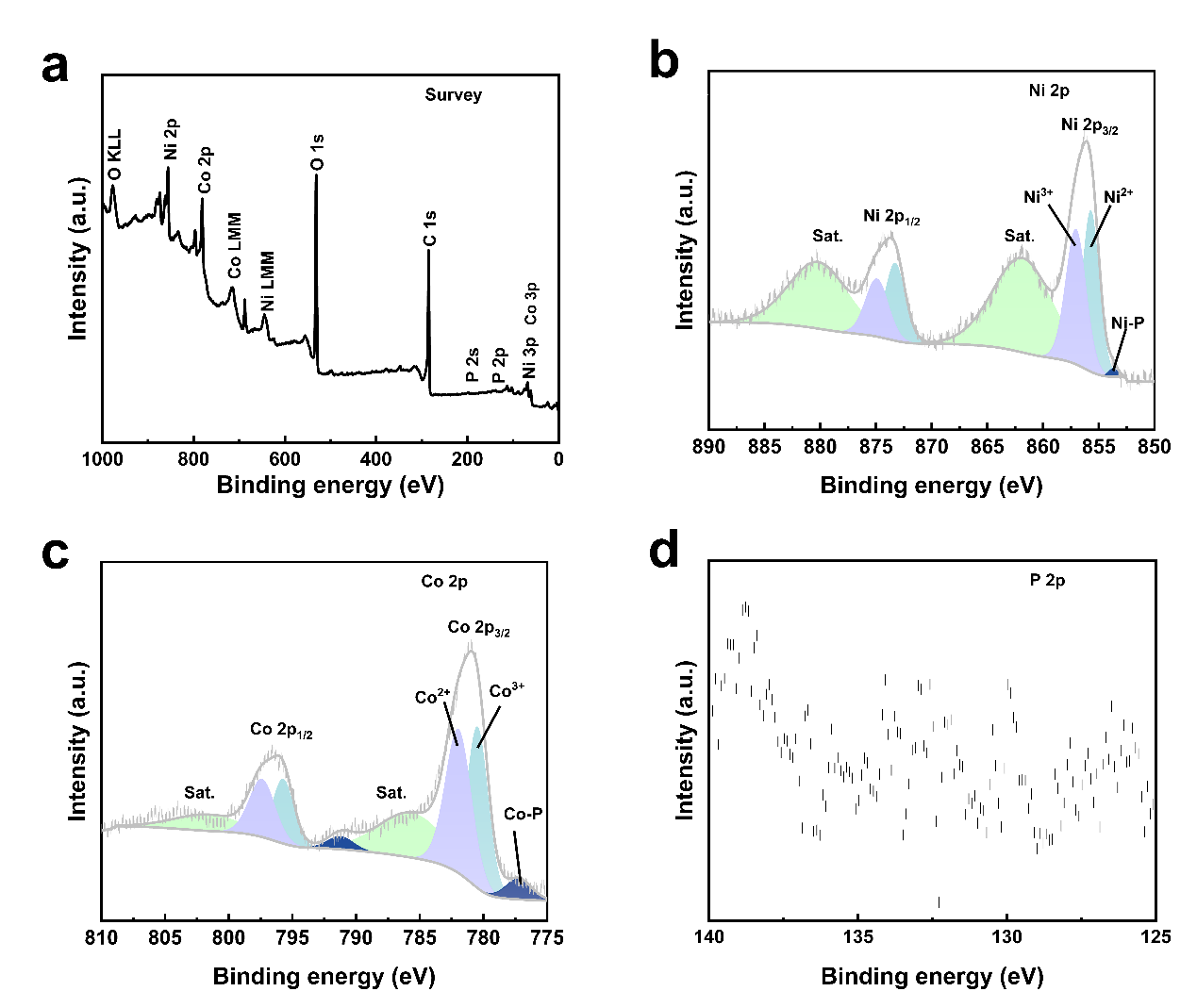


**Fig. S9** **a** XPS survey spectrum and high-resolution **b** Ni *2p*, **c** Co *2p*, and **d** P *2p* XPS spectra of NiCo-P1.0 after EA


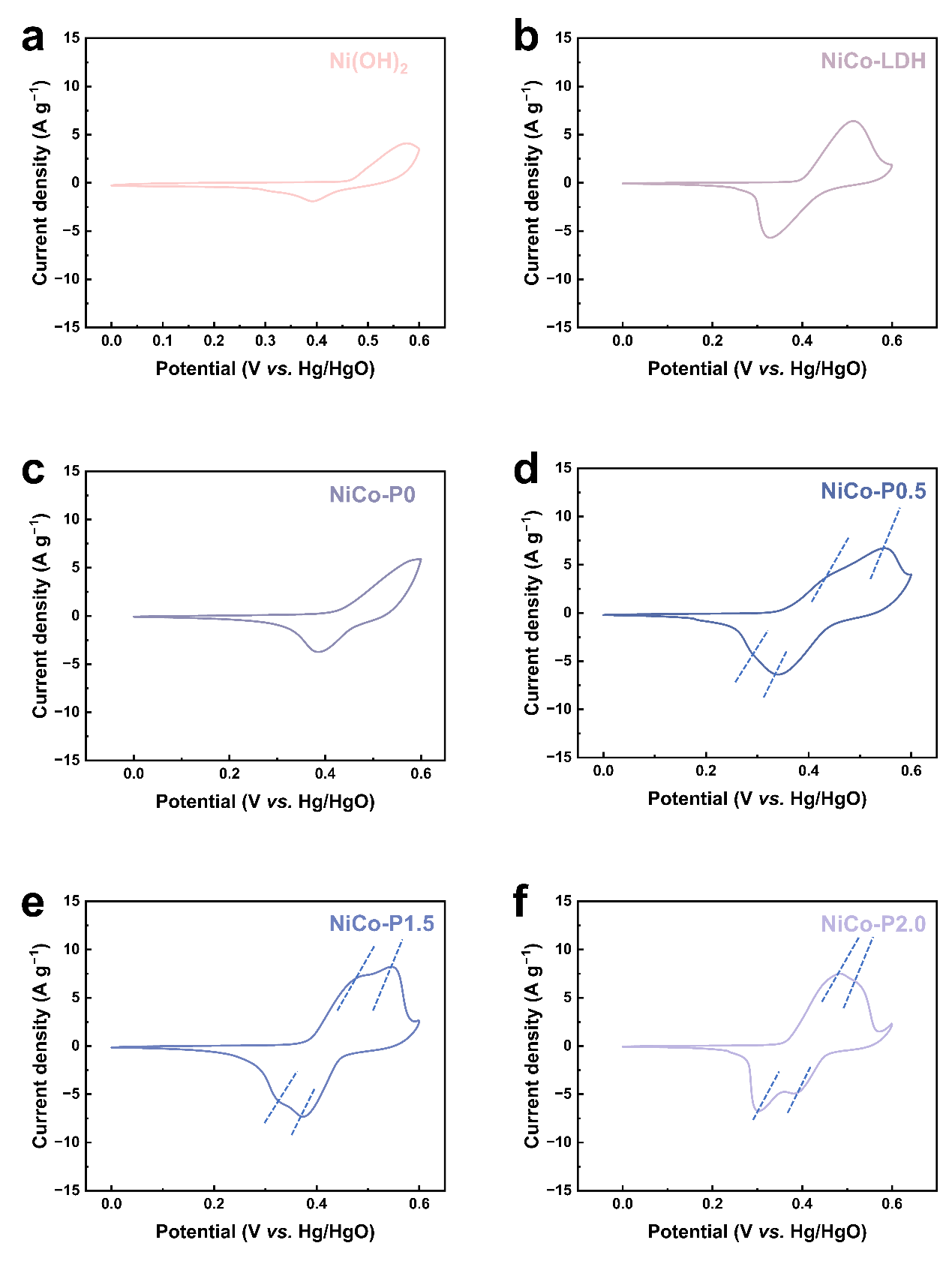


**Fig. S10** CV curves of **a** Ni(OH)_2_, **b** NiCoLDH, **c** NiCo-P0, **d** NiCo-P0.5, **e** NiCo-P1.5, and **f** NiCo-P2.0 at a scan rate of 1 mV s^−1^ in 3 mol L^−1^ KOH aqueous solution


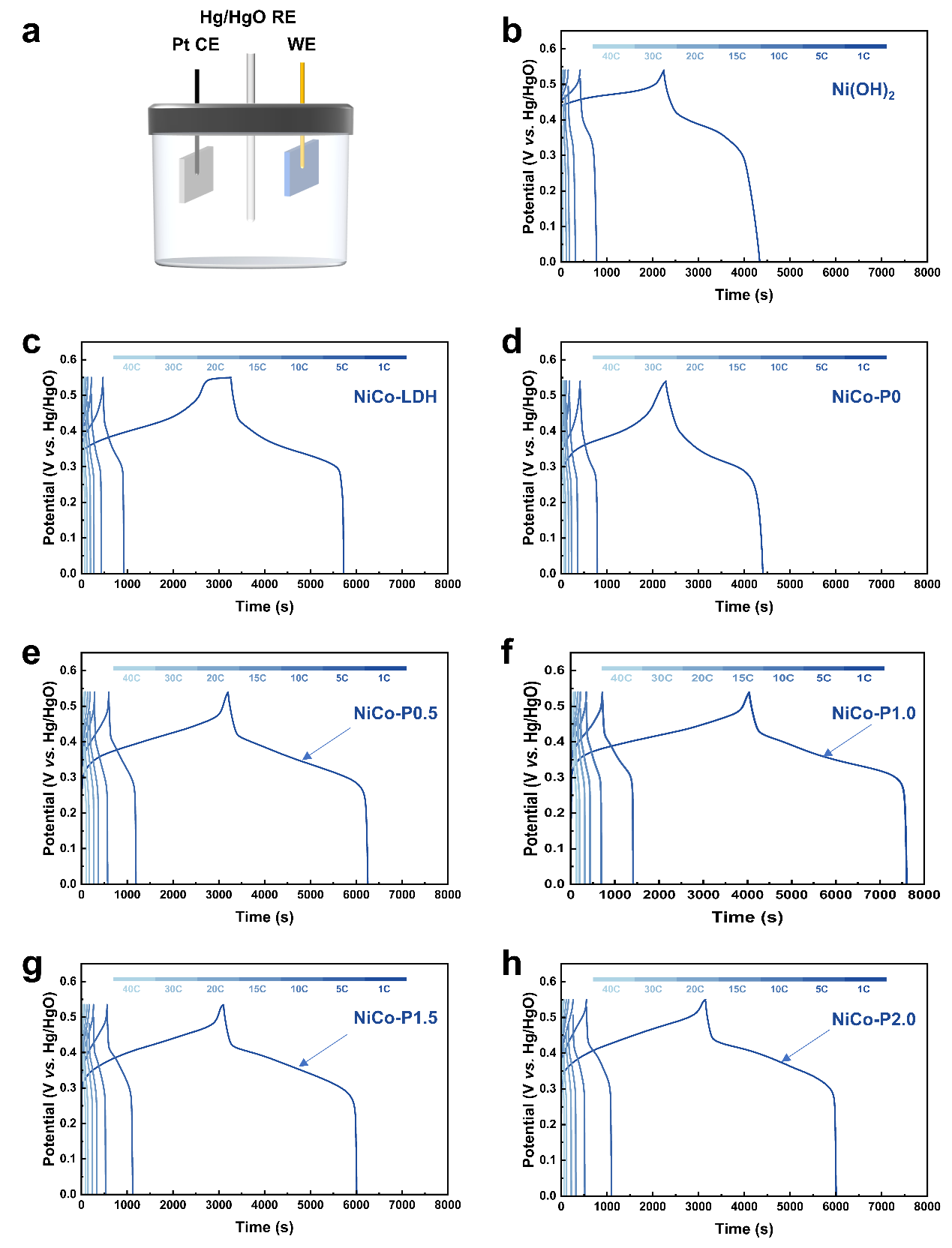


**Fig. S11 a** Schematic diagram of the three-electrode system. GCD curves of **b** Ni(OH)_2_, **c** NiCoLDH, **d** NiCo-P0, **e** NiCo-P0.5, **f** NiCo-P1.0, **g** NiCo-P1.5, and **h** NiCo-P2.0 at various current densities in 3 mol L^−1^ KOH aqueous solution


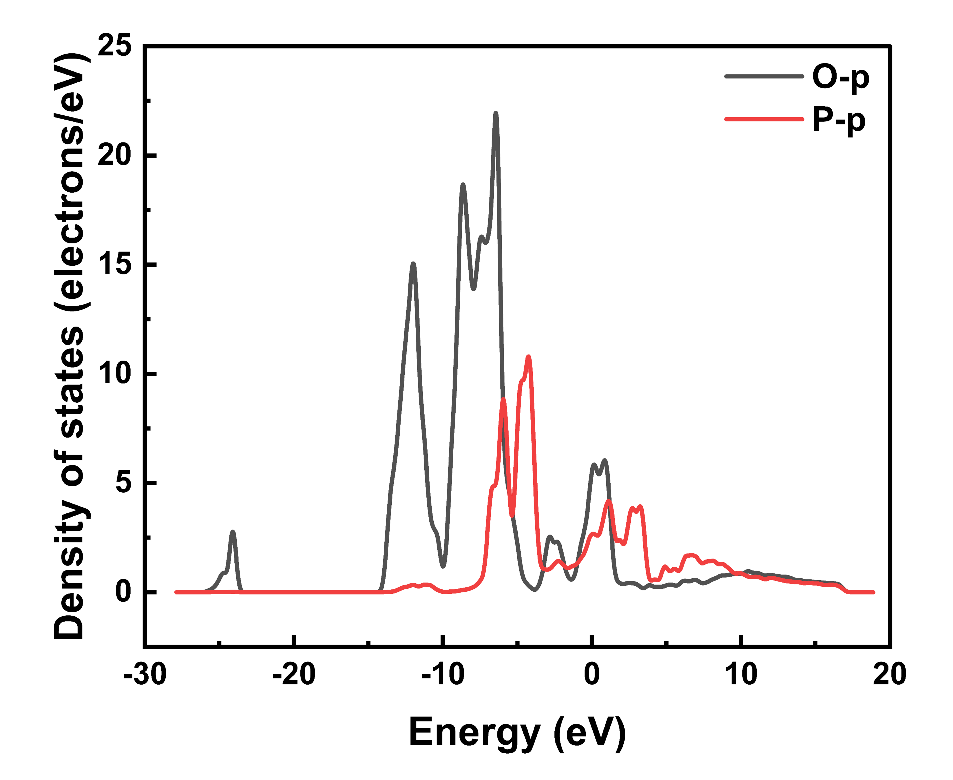


**Fig. S12** DOS of O and P of NiCo-P1.0


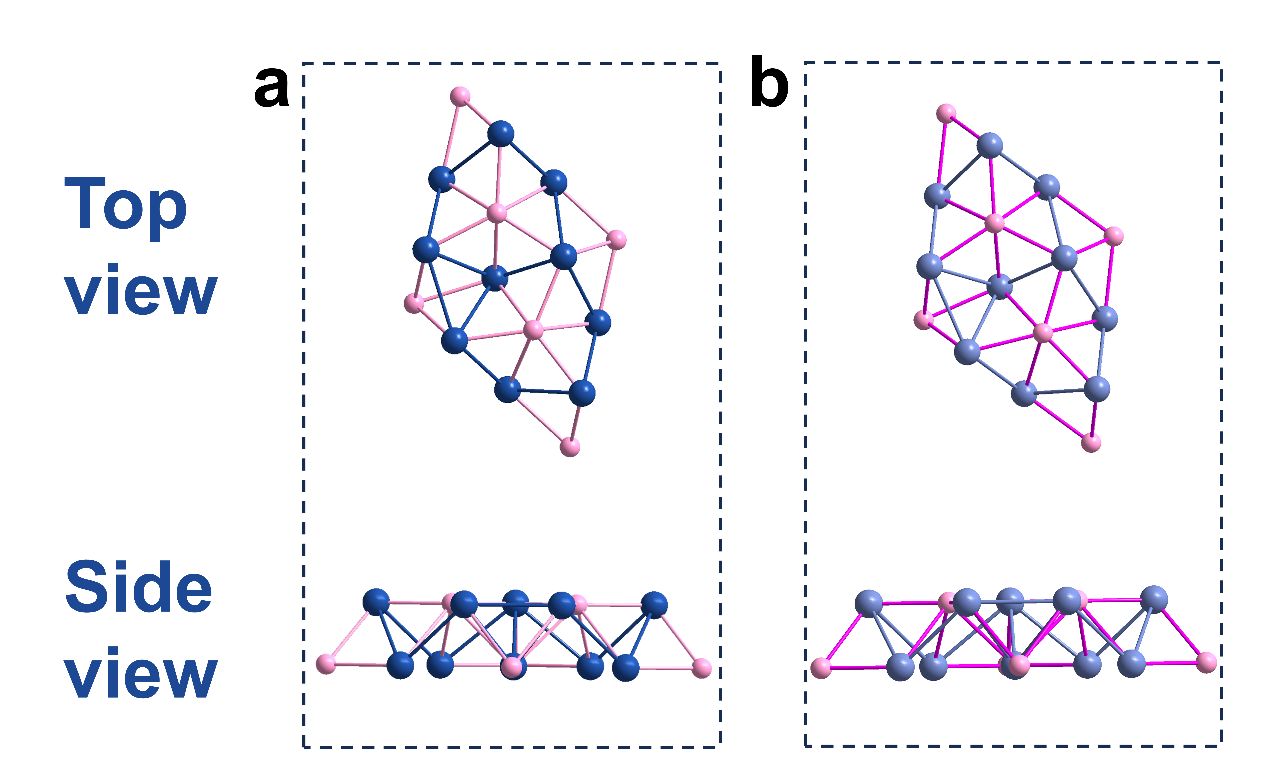


**Fig. S13** Optimized atomic structure models of **a** Ni_2_P and **b** Co_2_P


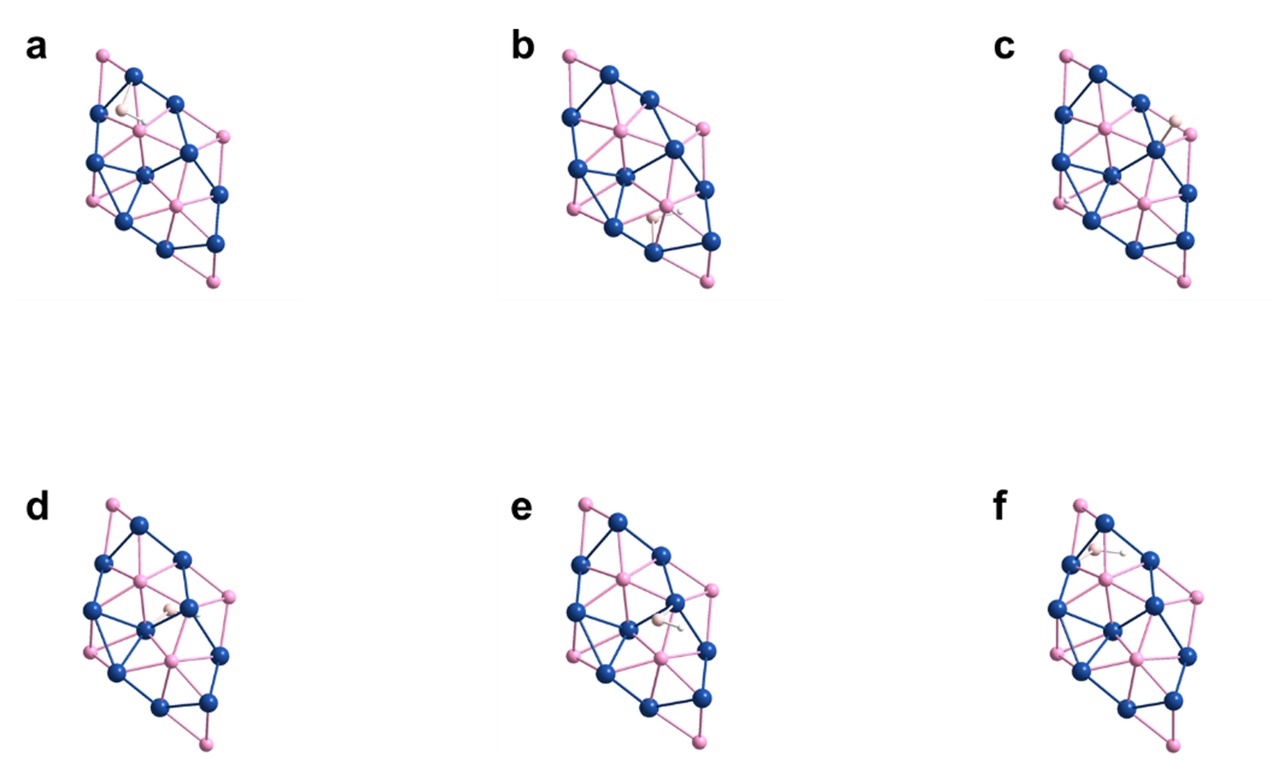


**Fig. S14** Optimized atomic structure models of Ni_2_P(OH) formed by OH⁻ binding to different sites on Ni_2_P. **a** Ni_2_P(OH)-1, **b** Ni_2_P(OH)-2, **c** Ni_2_P(OH)-3, **d** Ni_2_P(OH)-4, **e** Ni_2_P(OH)-5, and **f** Ni_2_P(OH)-6


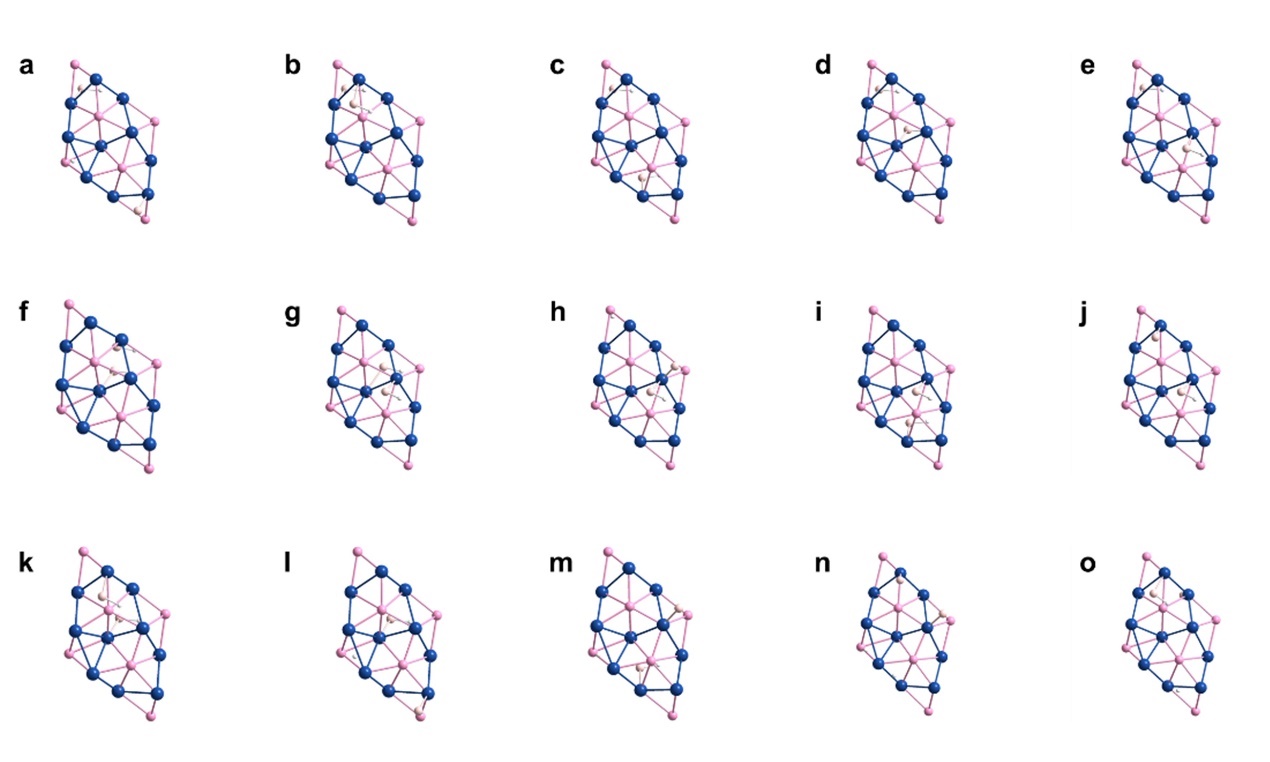


**Fig. S15** Optimized atomic structure models of Ni_2_P(OH)_2_ formed by OH⁻ binding to different sites on Ni_2_P. **a** Ni_2_P(OH)_2_-1, **b** Ni_2_P(OH)_2_-2, **c** Ni_2_P(OH)_2_-3, **d** Ni_2_P(OH)_2_-4, **e** Ni_2_P(OH)_2_-5, **f** Ni_2_P(OH)_2_-6, **g** Ni_2_P(OH)_2_-7, **h** Ni_2_P(OH)_2_-8, **i** Ni_2_P(OH)_2_-9, **j** Ni_2_P(OH)_2_-10, **k** Ni_2_P(OH)_2_-11, **l** Ni_2_P(OH)_2_-12, **m** Ni_2_P(OH)_2_-13, **n** Ni_2_P(OH)_2_-14, and **o** Ni_2_P(OH)_2_-15


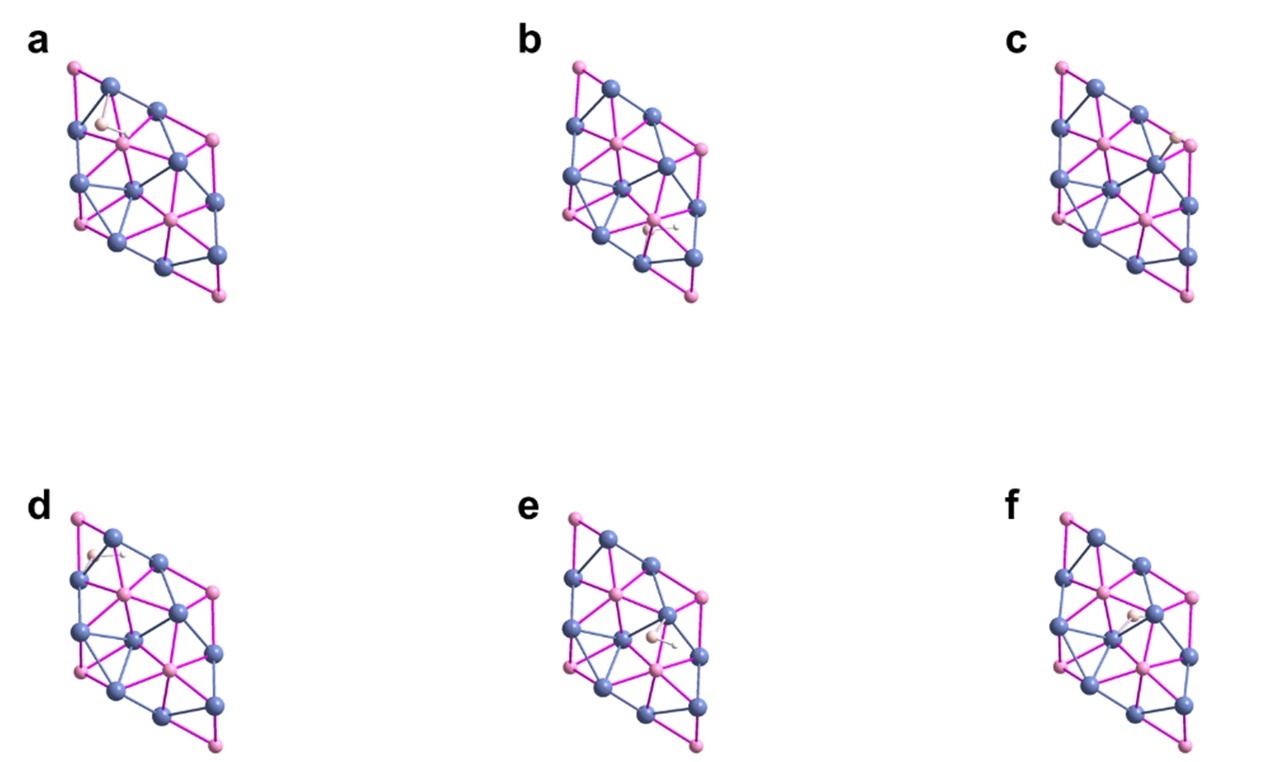


**Fig. S16** Optimized atomic structure models of Co_2_P(OH) formed by OH⁻ binding to different sites on Co_2_P. **a** Co_2_P(OH)-1, **b** Co_2_P(OH)-2, **c** Co_2_P(OH)-3, **d** Co_2_P(OH)-4, **e** Co_2_P(OH)-5, and **f** Co_2_P(OH)-6


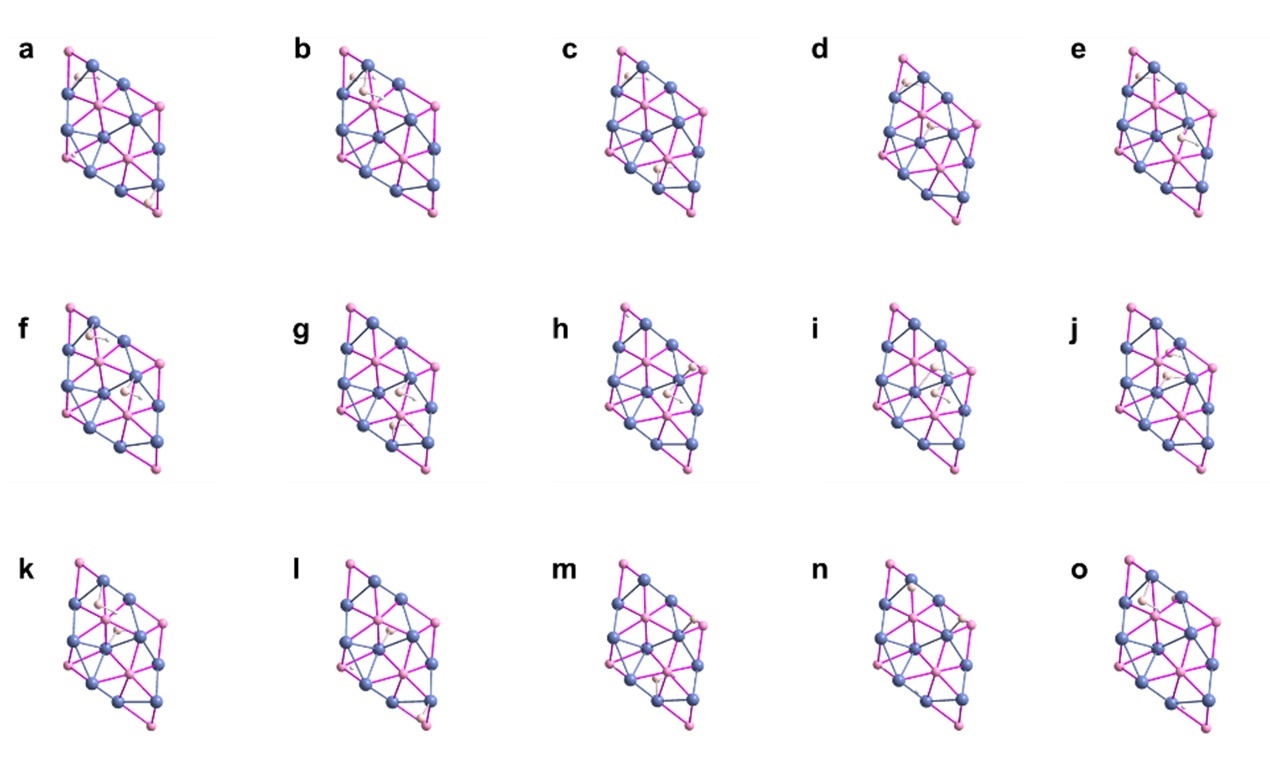


**Fig. S17** Optimized atomic structure models of Co_2_P(OH)_2_ formed by OH⁻ binding to different sites on Co_2_P. **a** Co_2_P(OH)_2_-1, **b** Co_2_P(OH)_2_-2, **c** Co_2_P(OH)_2_-3, **d** Co_2_P(OH)_2_-4, **e** Co_2_P(OH)_2_-5, **f** Co_2_P(OH)_2_-6, **g** Co_2_P(OH)_2_-7, **h** Co_2_P(OH)_2_-8, **i** Co_2_P(OH)_2_-9, **j** Co_2_P(OH)_2_-10, **k** Co_2_P(OH)_2_-11, **l** Co_2_P(OH)_2_-12, **m** Co_2_P(OH)_2_-13, **n** Co_2_P(OH)_2_-14, and **o** Co_2_P(OH)_2_-15


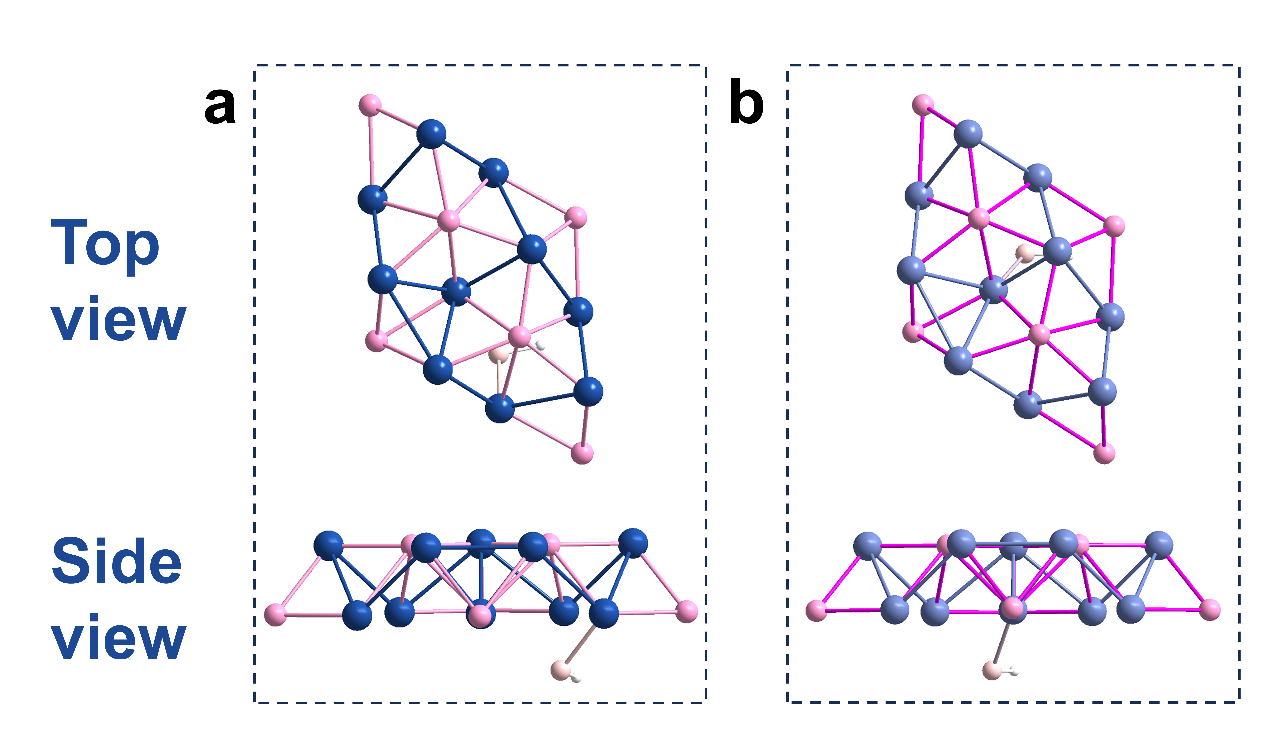


**Fig. S18** Optimized atomic structure with the lowest total energy of the theoretical calculation of **a** Ni_2_P(OH) and **b** Co_2_P(OH)


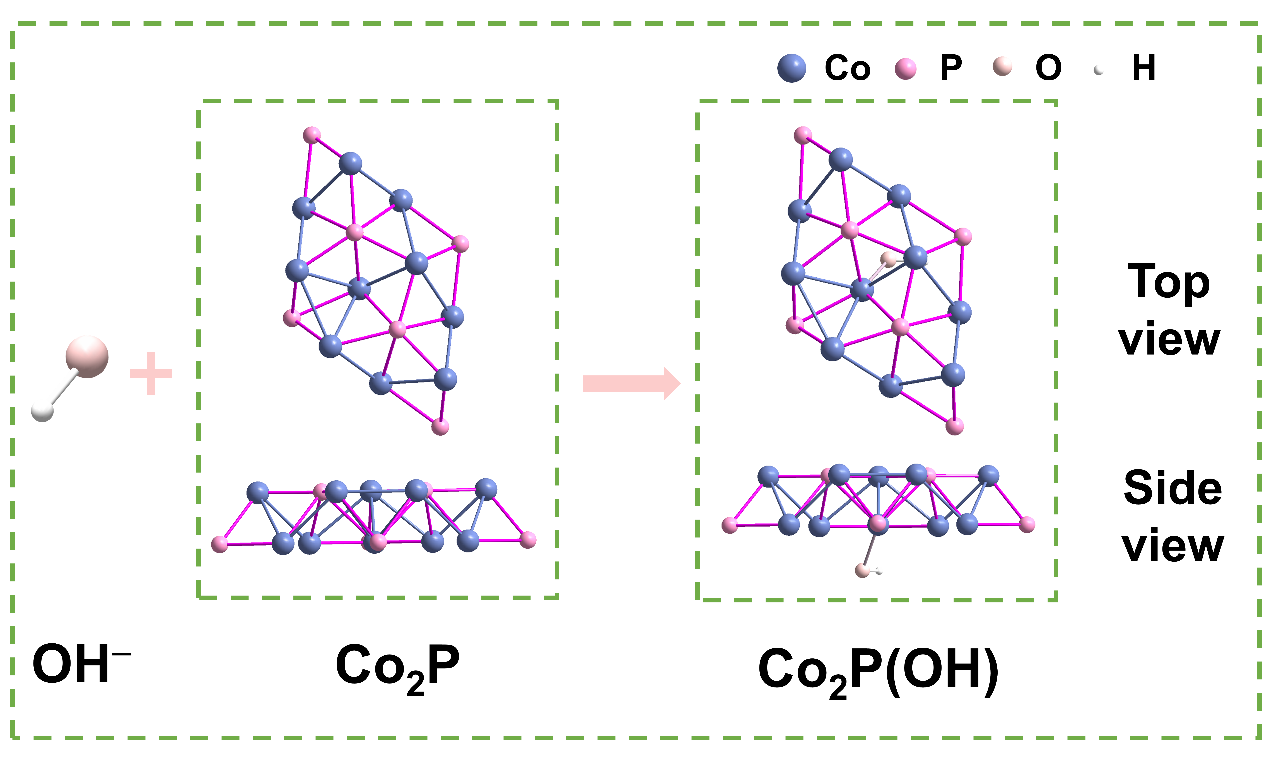


**Fig. S19** Schematic of the formation of Co_2_P(OH) by OH^−^ and Co_2_P


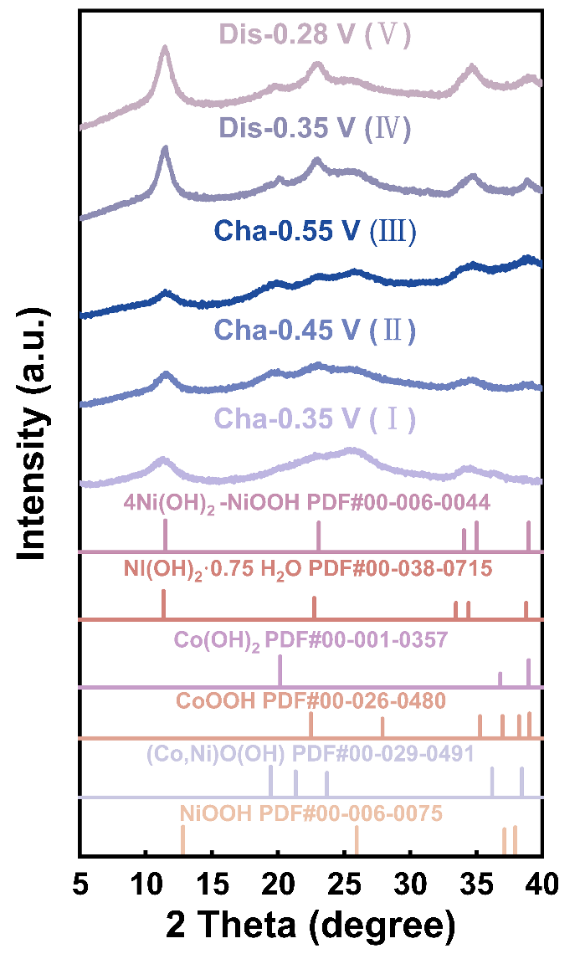


**Fig. S20** Ex situ XRD patterns collected during the whole charge/discharge process of NiCo-P1.0


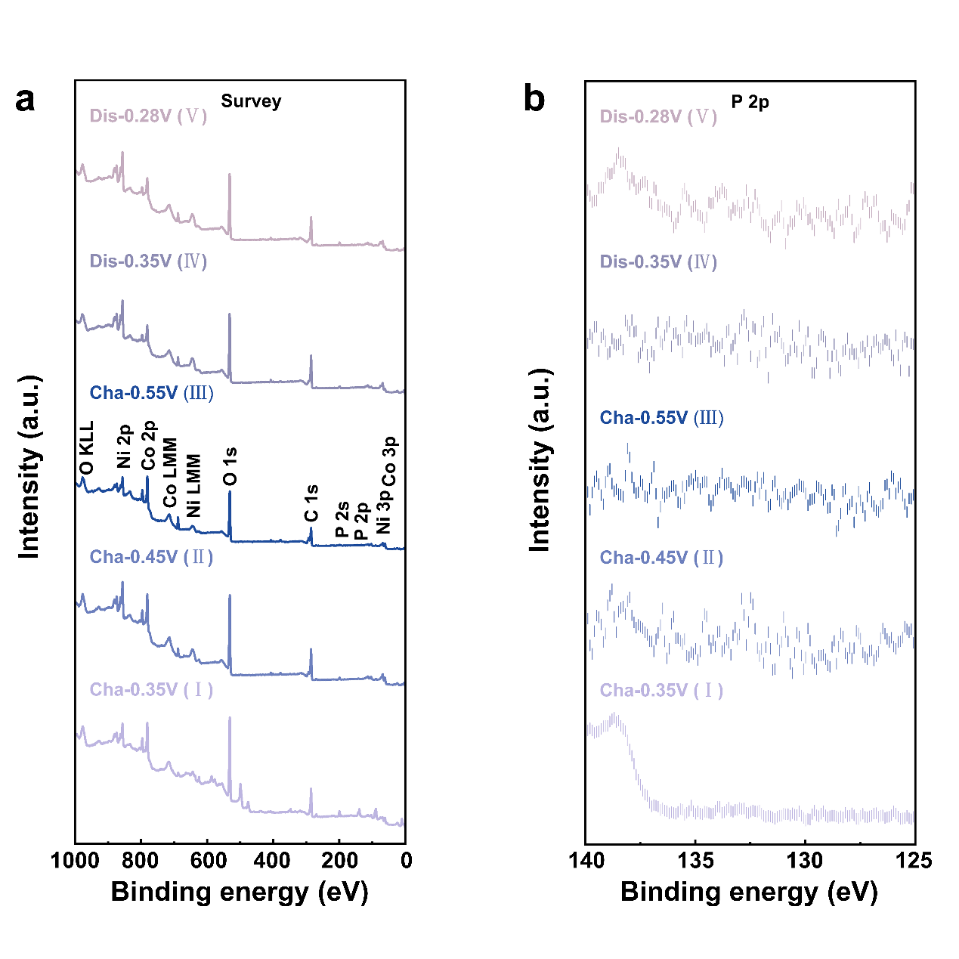


**Fig. S21** **a** XPS survey spectra and high-resolution. **b** P 2p at various charged/discharged states


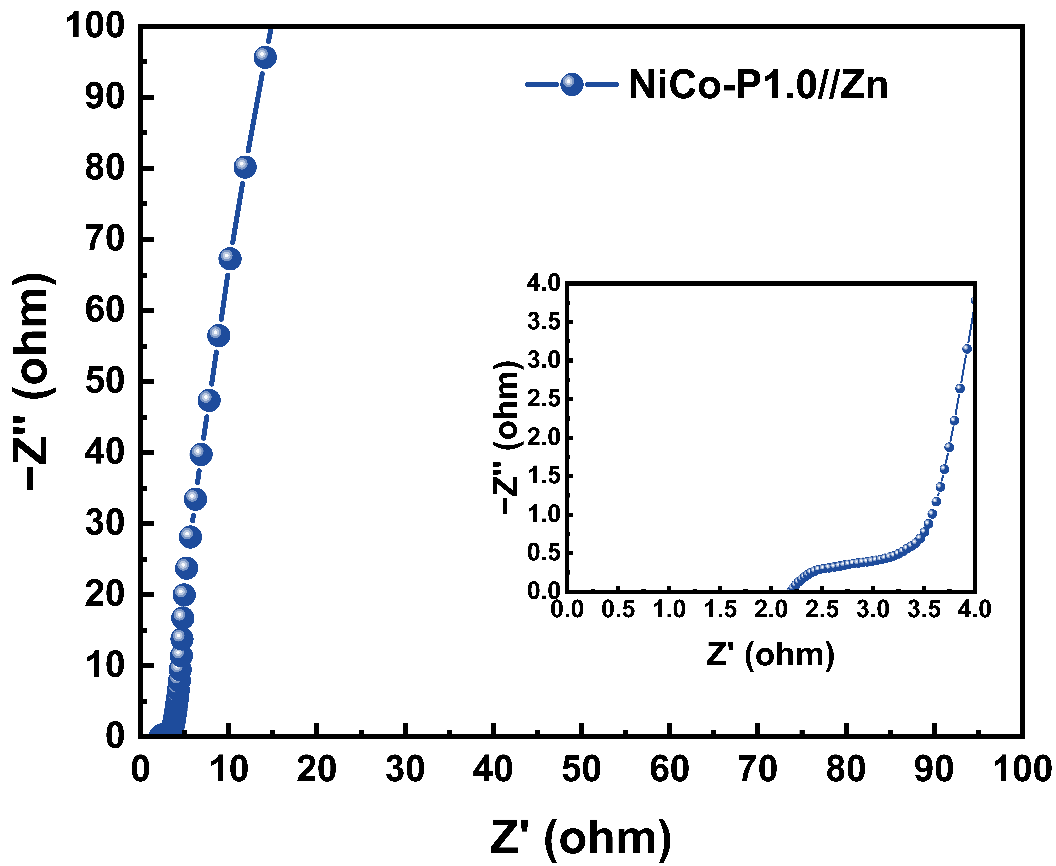


**Fig. S22** Nyquist plots of the NiCo-P1.0//Zn battery. The inset shows the plot at the high-frequency region


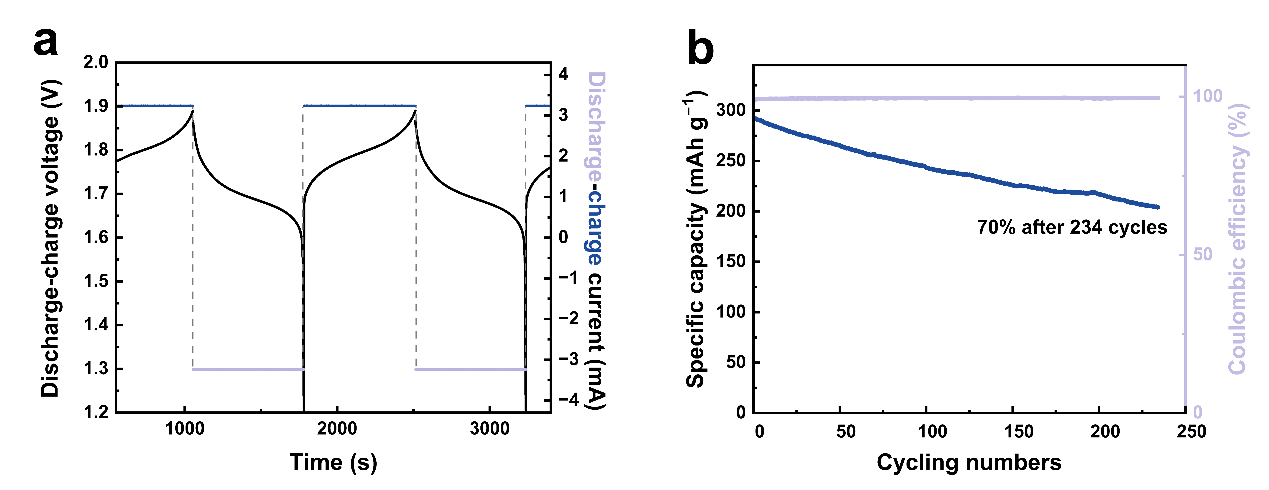


**Fig. S23** **a** The typical GCD curves of NiCo-P1.0//Zn battery at 5C. **b** The cycling performance of the NiCo-P1.0//Zn battery at 5C


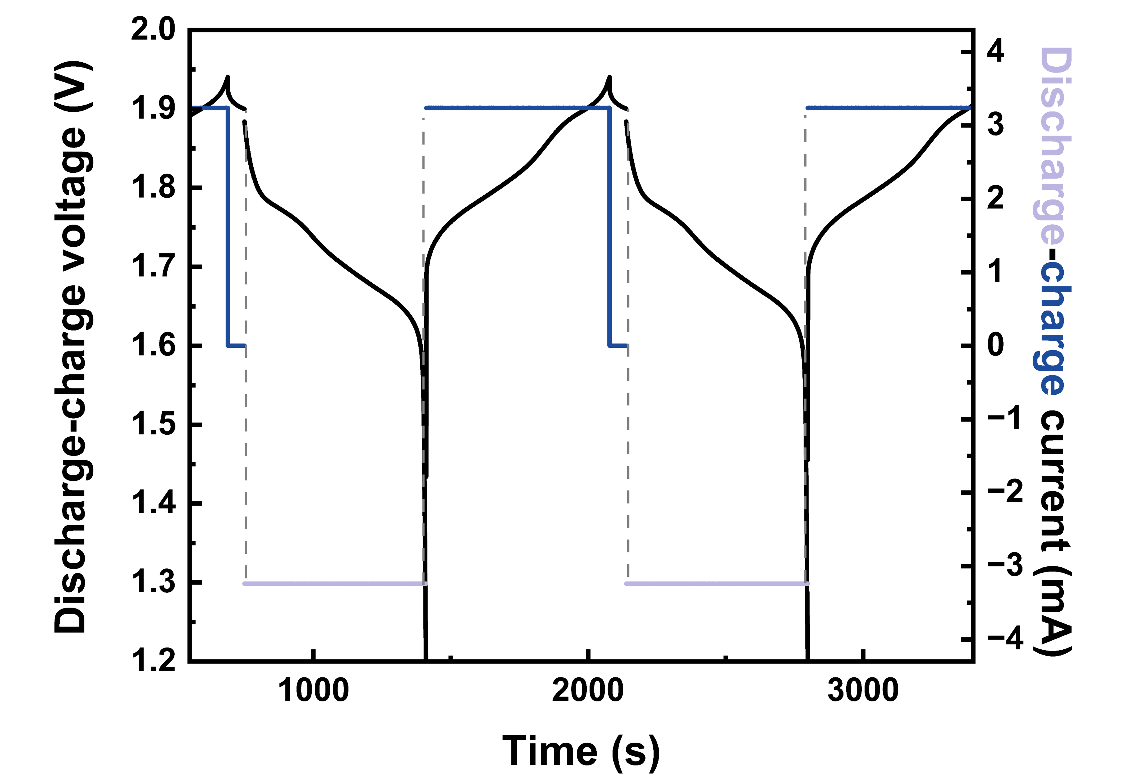


**Fig. S24** The voltage-time curves of NiCo-P1.0//Zn battery discharged and recharged at 5C. The constant voltage at 1.9 V indicating the potentiostatic region of the charge profile


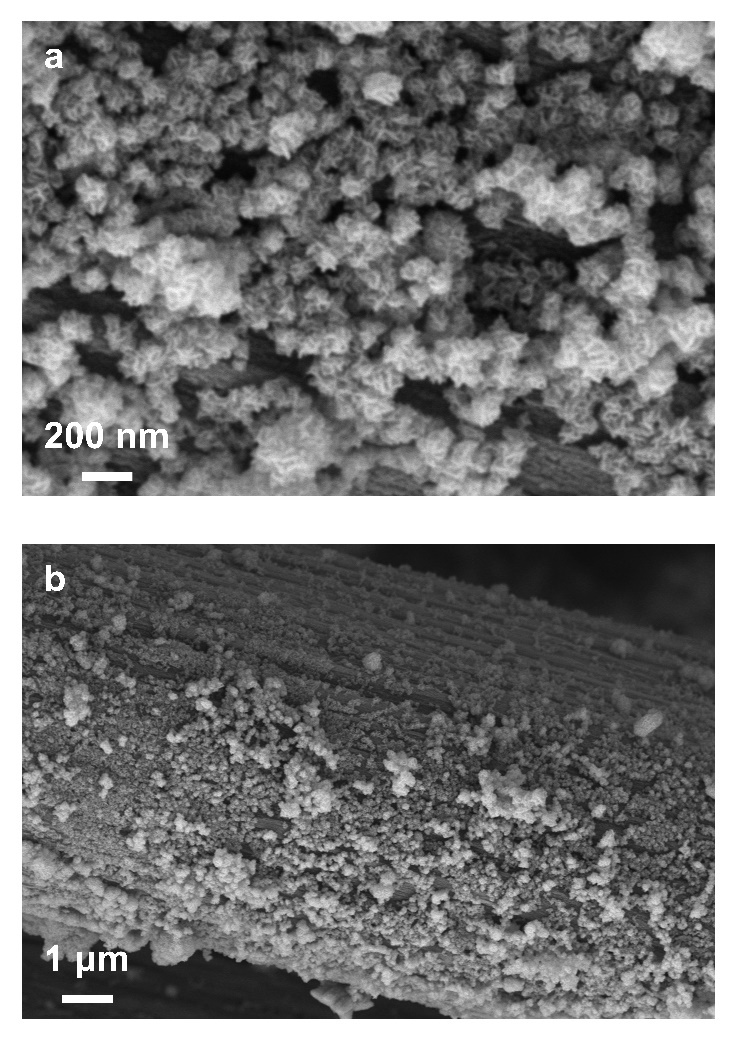


**Fig. S25** SEM images of NiCo-P1.0 after cycling


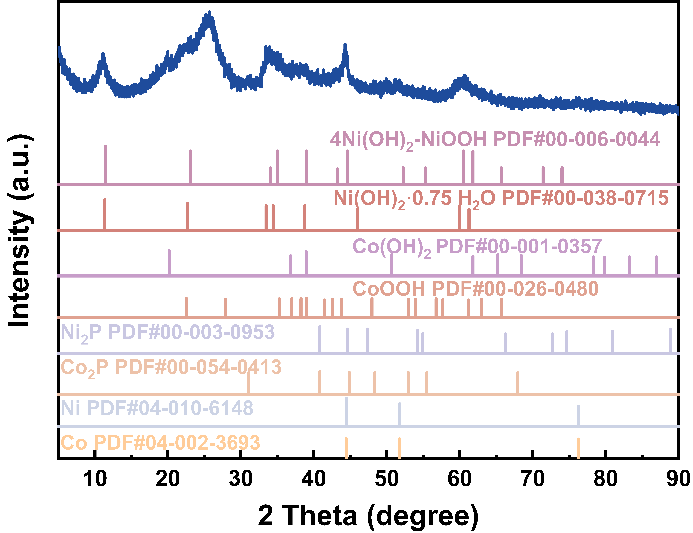


**Fig. S26** XRD patterns of the NiCo-P1.0 after cycling


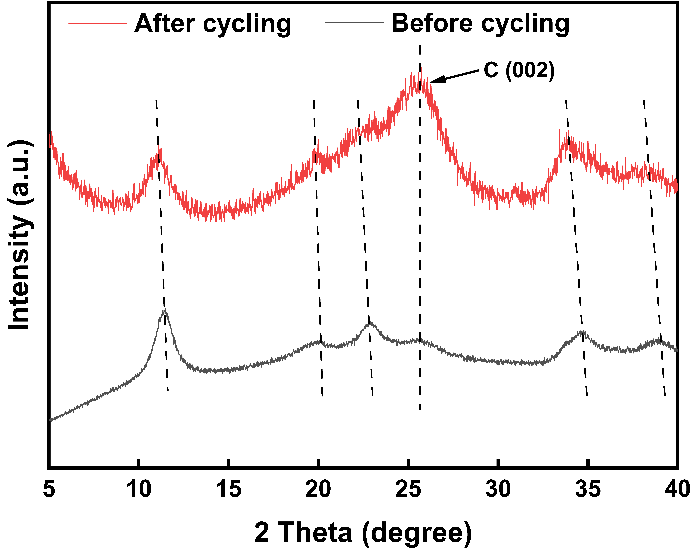


**Fig. S27** Comparison of XRD patterns of NiCo-P1.0 before and after cycling
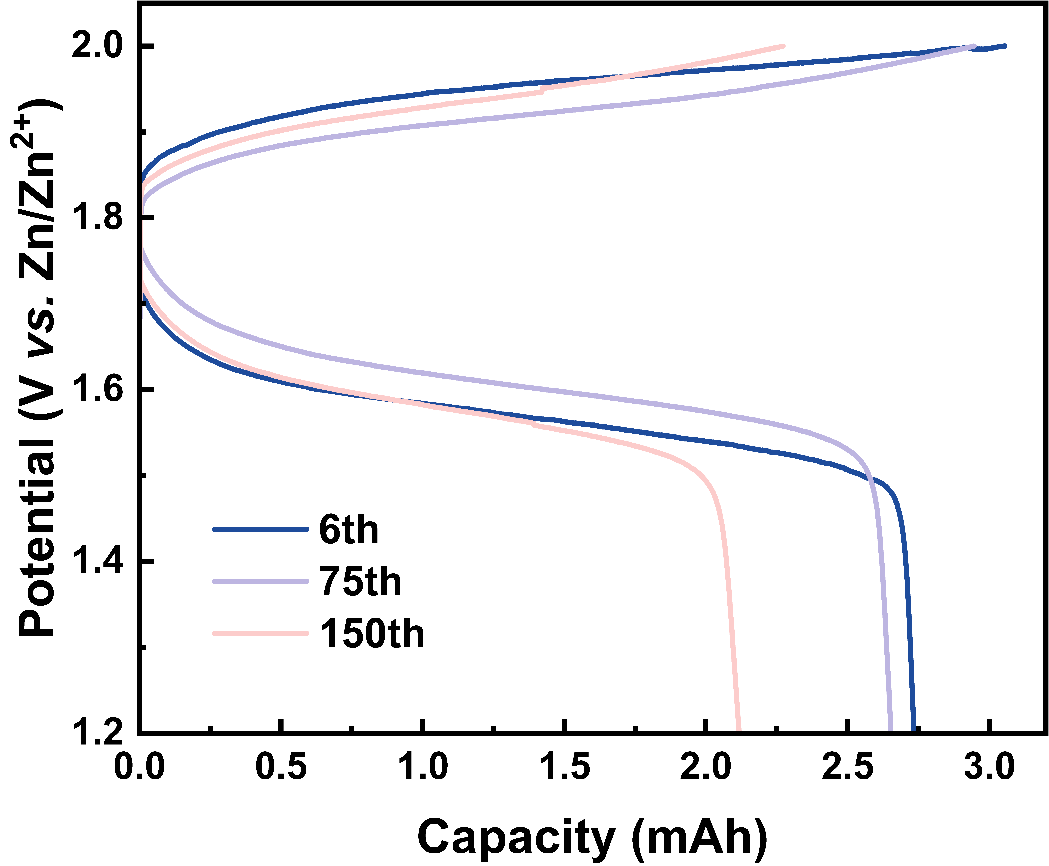


**Fig. S28** GCD curves of pouch cells with a size of 2 cm $\times$ 2 cm at different cycles

**Table S1** Representative nickel-based cathodes in ZBs

| **Cathode** | **Specific capacity** | **Cycling**  **Performance** |
| --- | --- | --- |
| (Ni, Co)Se_2_/NiCo-LDH  [S1] | 170 mAh g^−1^ at 2 A g^−1^,  capacity retention of 76% at 20 A g^−1^ | Capacity retention of 89.5% over 3000 cycles at 5 A g^−1^ |
| ZnCo30-Ni  [S2] | 213.3 mAh g^−1^ at 0.5 mA cm^−2^,  capacity retention of 81% at 20 mA cm^−2^ | Capacity retention of 76.2% over 5000 cycles at 30 mA cm^−2^ |
| Ni-Co microwire  [S3] | 0.723 mAh cm^−2^ at 0.5 mA cm^−2^,  capacity retention of 72% at 200 mA cm^−2^ | Capacity retention of 97.0% over 3000 cycles at 50 mA cm^−2^ |
| Ni-Ni(OH)_2_/Zn(OH)_2_  [S4] | 0.149 mAh cm^−2^ at 0.5 mA cm^−2^,  capacity retention of 86% at 200 mA cm^−2^ | Capacity retention of 91.2% over 3500 cycles at 20 mA cm^−2^ |
| NF/CH-MNCS  [S5] | 167.0 mAh g^−1^ at 2 mA cm^−2^,  capacity retention of 85% at 20 mA cm^−2^ | Capacity retention of 74% over 1000 cycles at 20 mA cm^−2^ |
| NiS-coated Ni_0.95_Zn_0.05_(OH)_2_  [S6] | 266.6 mAh g^−1^ at 22.4 mA cm^−2^,  capacity retention of 84% at 448 mA cm^−2^ | Capacity retention of 79% over 3500 cycles at 224 mA cm^−2^ |
| CoNiO_2_  [S7] | 180.20 mAh g^−1^ at 0.5 A g^−1^,  capacity retention of 58% at 5 A g^−1^ | Capacity retention of 103% over 5000 cycles at 5 A g^−1^ |
| NiMoO_4_-NC  [S8] | 221.0 mAh g^−1^ at 3.4 A g^−1^,  capacity retention of 53% at 8.6 A g^−1^ | Capacity retention of 89% over 3000 cycles at 6.4 A g^−1^ |
| MOF-74(Ni_0.675_Co_0.325_)-8  [S9] | 218.6 mAh g^−1^ at 2 mA cm^−2^,  capacity retention of 43% at 50 mA cm^−2^ | Capacity retention of 91% over 400 cycles at 10 mA cm^−2^ |

**Table S2** The details of Ni(OH)_2_, NiCo-LDH, and NiCo-P*x* (when *x* = 0, 0.5, 1.0, 1.5, 2.0) cathodes.

| **Cathode** | **Area of cathode**  **(cm^2^)** | **Mass loading of active material (mg cm^−2^)** |
| --- | --- | --- |
| Ni(OH)_2_ | 1.05 | 2.18 |
| NiCo-LDH | 1.13 | 2.14 |
| NiCo-P0 | 0.93 | 2.04 |
| NICo-P0.5 | 0.98 | 1.92 |
| NICo-P1.0 | 0.96 | 2.14 |
| NiCo-P1.5 | 0.91 | 1.83 |
| NiCo-P2.0 | 1.04 | 1.94 |

**Note:** Ni(OH)_2_, NiCo-LDH, and NiCo-P*x* (when *x* = 0, 0.5, 1.0, 1.5, 2.0) are all uniformly coated on the surface of the same type of carbon cloth current collector. Therefore, under ideal conditions, the electrode thickness should be consistent under the same mass loading.

**Table S3** Electrochemical performance of different samples in a three-electrode system.

| **Samples** | **Specific capacity**  **at 1C (mAh g^−1^)** | **Specific capacity**  **at 5C (mAh g^−1^)** | **Specific capacity**  **at 10C (mAh g^−1^)** | **Specific capacity**  **at 15C (mAh g^−1^)** | **Specific capacity**  **at 20C (mAh g^−1^)** | **Specific capacity**  **at 30C (mAh g^−1^)** | **Specific capacity**  **at 40C (mAh g^−1^)** |
| --- | --- | --- | --- | --- | --- | --- | --- |
| Ni(OH)_2_ | 168.18 | 143.18 | 125.88 | 109.50 | 93.22 | 48.99 | 11.55 |
| NiCo-LDH | 198.05 | 184.26 | 172.19 | 164.13 | 156.00 | 139.76 | 117.77 |
| NiCo-P0 | 169.80 | 150.43 | 141.04 | 134.29 | 128.53 | 117.20 | 103.63 |
| NiCo-P0.5 | 244.74 | 238.00 | 229.01 | 222.51 | 215.54 | 200.13 | 174.23 |
| NiCo-P1.0 | 286.64 | 279.68 | 271.10 | 262.31 | 253.53 | 233.98 | 207.00 |
| NiCo-P1.5 | 233.10 | 223.77 | 212.20 | 203.63 | 195.06 | 174.82 | 148.61 |
| NiCo-P2.0 | 228.78 | 217.92 | 205.66 | 191.98 | 177.12 | 143.81 | 109.33 |

**Table S4** Comparison of the binding energy of the models of Ni_2_P(OH) formed by OH⁻ binding to different sites on Ni_2_P

| **Model** | **Binding energy (kcal mol^−1^)** |
| --- | --- |
| Ni_6_P_3_ | - |
| OH^−^ | - |
| Different models of Ni_2_P(OH) |  |
| Ni_2_P(OH)-1 | −245 |
| Ni_2_P(OH)-2 | **−345** |
| Ni_2_P(OH)-3 | −261 |
| Ni_2_P(OH)-4 | −343 |
| Ni_2_P(OH)-5 | −261 |
| Ni_2_P(OH)-6 | −333 |
| Different models of Ni_2_P(OH)_2_ |  |
| Ni_2_P(OH)_2_-1 | 144 |
| Ni_2_P(OH)_2_-2 | 144 |
| Ni_2_P(OH)_2_-3 | 55 |
| Ni_2_P(OH)_2_-4 | 110 |
| Ni_2_P(OH)_2_-5 | 147 |
| Ni_2_P(OH)_2_-6 | 230 |
| Ni_2_P(OH)_2_-7 | 142 |
| Ni_2_P(OH)_2_-8 | 241 |
| Ni_2_P(OH)_2_-9 | 137 |
| Ni_2_P(OH)_2_-10 | 113 |
| Ni_2_P(OH)_2_-11 | 140 |
| Ni_2_P(OH)_2_-12 | 138 |
| Ni_2_P(OH)_2_-13 | 142 |
| Ni_2_P(OH)_2_-14 | 243 |
| Ni_2_P(OH)_2_-15 | 139 |

**Table S5** Comparison of the binding energy of the models of Co_2_P(OH) formed by OH⁻ binding to different sites on Co_2_P.

| **Model** | **Binding energy**  **(kcal mol^−1^)** |
| --- | --- |
| Co_6_P_3_ | - |
| OH^−^ | - |
| Different models of Co_2_P(OH) |  |
| Co_2_P(OH)-1 | −623 |
| Co_2_P(OH)-2 | −701 |
| Co_2_P(OH)-3 | −609 |
| Co_2_P(OH)-4 | **−702** |
| Co_2_P(OH)-5 | −622 |
| Co_2_P(OH)-6 | −698 |
| Different models of Co_2_P(OH)_2_ |  |
| Co_2_P(OH)_2_-1 | −196 |
| Co_2_P(OH)_2_-2 | −237 |
| Co_2_P(OH)_2_-3 | −327 |
| Co_2_P(OH)_2_-4 | −277 |
| Co_2_P(OH)_2_-5 | −233 |
| Co_2_P(OH)_2_-6 | −145 |
| Co_2_P(OH)_2_-7 | −239 |
| Co_2_P(OH)_2_-8 | −120 |
| Co_2_P(OH)_2_-9 | −246 |
| Co_2_P(OH)_2_-10 | −308 |
| Co_2_P(OH)_2_-11 | −242 |
| Co_2_P(OH)_2_-12 | −243 |
| Co_2_P(OH)_2_-13 | −219 |
| Co_2_P(OH)_2_-14 | −140 |
| Co_2_P(OH)_2_-15 | −239 |

**Table S6** Comparison of some representative NZBs (energy and power densities are based on the active material’s mass loading of the cathode).

| **Battery** | **Maximum energy density (Wh kg^−1^)** | **Maximum power density (kW kg^−1^)** | **Reference** |
| --- | --- | --- | --- |
| NCO-Ar//Zn | 308.14 | 8.52 | [S7] |
| NiMoO_4_-NC//Zn | 407.8 | 18.1 | [S8] |
| MOF-74(Ni_0.675_Co_0.325_)-8//Zn | 266.5 | 17.22 | [S9] |
| Ni-mMeSA//Zn@CC | 125 | 14.03 | [S10] |
| NZMCO//Zn | 120.4 | 1.88 | [S11] |
| CoNi LDH(v)//Zn | 296.2 | 18.5 | [S12] |
| sd-NiCo_2_S_4_-*x*@CC-1.2 g Na_2_S//Zn | 469.6 | 7.343 | [S13] |
| NiCo-P1.0//Zn | 503.62 | 18.61676 | This work |

**Table S7** Comparison of this work with some representative cathodes in ZBs

| **Cathode** | **Specific capacity**  **(**mAh g^−1^**)** | **Cycling**  **performance** |
| --- | --- | --- |
| K_2_MnFe(CN)_6_  [S14] | 138 | Capacity retention of 72% over 400 cycles at 0.20 A g^−1^ |
| Zn_3_[Fe(CN)_6_]_2_  [S15] | 65 | Capacity retention of 81% over 100 cycles at 0.30 A g^−1^ |
| Na_2_MnFe(CN)_6_  [S16] | 137 | Capacity retention of 75% over 2000 cycles at 0.80 A g^−1^ |
| NiHCF  [S17] | 76 | Capacity retention of 81% over 1000 cycles at 0.50 A g^−1^ |
| ZnHCF  [S18] | 95 | Capacity retention of 88% over 300 cycles at 0.25 A g^−1^ |
| Cu[Fe(CN)_6_]_2/3_∙nH_2_O  [S19] | 60 | Capacity retention of 96% over 50 cycles at 0.05 A g^−1^ |
| MnO_2_@AEPA  [S20] | 223 | Capacity retention of 97% over 1700 cycles at 1.00 A g^−1^ |
| α-MnO_2_  [S21] | 285 | Capacity retention of 92% over 5,000 cycles at 1.54 A g^−1^ |
| β-MnO_2_  [S22] | 225 | Capacity retention of 94% over 2000 cycles at 2.00 A g^−1^ |
| PANI-intercalated MnO_2_  [S23] | 280 | Capacity retention of 89% over 5000 cycles at 2.00 A g^−1^ |
| MnO_2_@N  [S24] | 183 | Capacity retention of 83% over 1000 cycles at 5.00 A g^−1^ |
| Zn_0.25_V_2_O_5_·nH_2_O  [S25] | 282 | Capacity retention of 81% over 1,000 cycles at 2.40 A g^−1^ |
| NaV_3_O_8_∙1.5H_2_O  [S26] | 380 | Capacity retention of 82% over 1,000 cycles at 4.00 A g^−1^ |
| MnVO  [S27] | 260 | Capacity retention of 96% over 2000 cycles at 4.00 A g^−1^ |
| VO_2_ (B) nanofibers  [S28] | 357 | Capacity retention of 91% over 300 cycles at 0.85 A g^−1^ |
| V_2_O_5_·nH_2_O  [S29] | 381 | Capacity retention of 71% over 900 cycles at 6.00 A g^−1^ |
| MoS_2_/PANI  [S30] | 107 | Capacity retention of 86% over 1000 cycles at 1.00 A g^−1^ |
| MoS_2_·nH_2_O  [S31] | 165 | Capacity retention of 88% over 800 cycles at 2.00 A g^−1^ |
| 200-MoS_2_  [S32] | 125 | Capacity retention of 100% over 500 cycles at 2.00 A g^−1^ |
| VS_2_@SS  [S33] | 198 | Capacity retention of 80% over 2000 cycles at 2.00 A g^−1^ |
| **Cathode** | **Specific capacity**  **(**mAh g^−1^**)** | **Cycling**  **performance** |
| VS_2_@N–C  [S34] | 203 | Capacity retention of 97% over 600 cycles at 1.00 A g^−1^ |
| rGO-VS_2_  [S35] | 238 | Capacity retention of 93% over 1000 cycles at 5.00 A g^−1^ |
| NiCo-P1.0  (This work) | 295 | Capacity retention of 70% over 1150 cycles at 1.45 A g^−1^ |

**S4 Supplementary Equations**

${5NaH}_{2}{PO}_{2}\to{2PH}_{3}+{2H}_{2}+{Na}_{3}{PO}_{4}+{2NaPO}_{3}$ (S9)

${2PH}_{3}\to2P+3H_{2}$ (S10)

${Ni(OH)}_{2}+H_{2}\to Ni+2H_{2}O$ (S11)

${Co(OH)}_{2}+H_{2}\to Co+2H_{2}O$ (S12)

$2Ni+P\to{Ni}_{2}P$ (S13)

$2Co+P\to{Co}_{2}P$ (S14)

$3{Ni}_{2}P+P+16H_{2}O\to{2Ni}_{3}{(PO}_{4})_{2}+16H_{2}$ (S15)

$3{Co}_{2}P+P+16H_{2}O\to{2Co}_{3}{(PO}_{4})_{2}+16H_{2}$ (S16)

${Ni}_{2}P+3P+12H_{2}O\to2Ni{(PO}_{3})_{2}+12H_{2}$ (S17)

${Co}_{2}P+3P+12H_{2}O\to2Co{(PO}_{3})_{2}+12H_{2}$ (S18)

**Supplementary References**

1. X. Li, H. Wu, C. Guan, A.M. Elshahawy, Y. Dong et al., (Ni, co)Se_2_/NiCo-LDH core/shell structural electrode with the *Cactus*-like (Ni, co)Se_2_ core for asymmetric supercapacitors. Small **15**(3), 1803895 (2019). <https://doi.org/10.1002/smll.201803895>
2. Y. Pang, L. Li, Y. Wang, X. Zhu, J. Ge et al., Zinc-induced phase reconstruction of cobalt–nickel double hydroxide cathodes for high-stability and high-rate nickel–zinc batteries. Chem. Eng. J. **436**, 135202 (2022). <https://doi.org/10.1016/j.cej.2022.135202>
3. L. Lv, Z. Zhu, X. Liao, L. Wu, Y. Duan et al., Deeply reconstructed hierarchical Ni-co microwire for flexible Ni-Zn microbattery with excellent comprehensive performance. Small **19**(36), e2301913 (2023). <https://doi.org/10.1002/smll.202301913>
4. Z. Zhu, R. Kan, P. Wu, Y. Ma, Z. Wang et al., A durable Ni-Zn microbattery with ultrahigh-rate capability enabled by *in situ* reconstructed nanoporous nickel with epitaxial phase. Small **17**(42), e2103136 (2021). <https://doi.org/10.1002/smll.202103136>
5. D. Cai, Y. Wang, B. Fei, C.L. Cheng, C. Zhang et al., Engineering of MoSe_2_ decorated Ni/Co selenide complex hollow arrayed structures with dense heterointerfaces for high-performance aqueous alkaline Zn batteries. Chem. Eng. J. **450**, 138341 (2022). <https://doi.org/10.1016/j.cej.2022.138341>
6. W. Zhou, D. Zhu, J. He, J. Li, H. Chen et al., A scalable top-down strategy toward practical metrics of Ni–Zn aqueous batteries with total energy densities of 165 W h kg−1 and 506 W h L−1. Energy Environ. Sci. **13**(11), 4157–4167 (2020). <https://doi.org/10.1039/D0EE01221A>
7. J. Ye, X. Zhai, L. Chen, W. Guo, T. Gu et al., Oxygen vacancies enriched nickel cobalt based nanoflower cathodes: Mechanism and application of the enhanced energy storage. J. Energy Chem. **62**, 252–261 (2021). <https://doi.org/10.1016/j.jechem.2021.03.030>
8. L. Zhou, S. Zeng, D. Zheng, Y. Zeng, F. Wang et al., NiMoO_4_ nanowires supported on Ni/C nanosheets as high-performance cathode for stable aqueous rechargeable nickel-zinc battery. Chem. Eng. J. **400**, 125832 (2020). <https://doi.org/10.1016/j.cej.2020.125832>
9. T. Chen, H. Xu, S. Li, J. Zhang, Z. Tan et al., Tailoring the electrochemical responses of MOF-74 *via* dual-defect engineering for superior energy storage. Adv. Mater. **36**(31), 2402234 (2024). <https://doi.org/10.1002/adma.202402234>
10. Y. Su, J. Hu, G. Yuan, G. Zhang, W. Wei et al., Regulating intramolecular electron transfer of nickel-based coordinations through ligand engineering for aqueous batteries. Adv. Mater. **35**(48), 2307003 (2023). <https://doi.org/10.1002/adma.202307003>
11. Z. Zhu, R. Zhang, J. Lin, K. Zhang, N. Li et al., Ni, Zn-codoped MgCo_2_O_4_ electrodes for aqueous asymmetric supercapacitor and rechargeable Zn battery. J. Power Sources **437**, 226941 (2019). <https://doi.org/10.1016/j.jpowsour.2019.226941>
12. J. Meng, Y. Song, Z. Qin, Z. Wang, X. Mu et al., Cobalt–nickel double hydroxide toward mild aqueous zinc-ion batteries. Adv. Funct. Mater. **32**(33), 2204026 (2022). <https://doi.org/10.1002/adfm.202204026>
13. C. Han, T. Zhang, J. Li, B. Li, and Z. Lin, Hierarchical NiCo₂S₄@MoS₂ core-shell nanowire arrays for high-performance flexible solid-state asymmetric supercapacitors. Nano Energy **77**, 105165 (2020). <https://doi.org/10.1016/j.nanoen.2020.105165>
14. W. Deng, Z. Li, Y. Ye, Z. Zhou, Y. Li et al., Zn^2+^ induced phase transformation of K_2_MnFe(CN)_6_ boosts highly stable zinc-ion storage. Adv. Energy Mater. **11**(31), 2003639 (2021). <https://doi.org/10.1002/aenm.202003639>
15. L. Zhang, L. Chen, X. Zhou, Z. Liu, Towards high-voltage aqueous metal-ion batteries beyond 1.5 V: the zinc/zinc hexacyanoferrate system. Adv. Energy Mater. **5**(2), 1400930 (2015). <https://doi.org/10.1002/aenm.201400930>
16. Z. Hou, X. Zhang, X. Li, Y. Zhu, J. Liang et al., Surfactant widens the electrochemical window of an aqueous electrolyte for better rechargeable aqueous sodium/zinc battery. J. Mater. Chem. A **5**(2), 730–738 (2017). <https://doi.org/10.1039/C6TA08736A>
17. K. Lu, B. Song, J. Zhang, H. Ma, A rechargeable Na-Zn hybrid aqueous battery fabricated with nickel hexacyanoferrate and nanostructured zinc. J. Power Sources **321**, 257–263 (2016). <https://doi.org/10.1016/j.jpowsour.2016.05.003>
18. Q. Zhang, C. Li, Q. Li, Z. Pan, J. Sun et al., Flexible and high-voltage coaxial-fiber aqueous rechargeable zinc-ion battery. Nano Lett. **19**(6), 4035–4042 (2019). <https://doi.org/10.1021/acs.nanolett.9b01403>
19. V. Renman, D.O. Ojwang, M. Valvo, C.P. Gómez, T. Gustafsson et al., Structural-electrochemical relations in the aqueous copper hexacyanoferrate-zinc system examined by synchrotron X-ray diffraction. J. Power Sources **369**, 146–153 (2017). <https://doi.org/10.1016/j.jpowsour.2017.09.079>
20. X. Xiao, L. Zhang, W. Xin, M. Yang, Y. Geng et al., Self-assembled layer of organic phosphonic acid enables highly stable MnO_2_ cathode for aqueous znic batteries. Small **20**(24), e2309271 (2024). <https://doi.org/10.1002/smll.202309271>
21. H. Pan, Y. Shao, P. Yan, Y. Cheng, K.S. Han et al., Reversible aqueous zinc/manganese oxide energy storage from conversion reactions. Nat. Energy **1**(5), 16039 (2016). <https://doi.org/10.1038/nenergy.2016.39>
22. N. Zhang, F. Cheng, J. Liu, L. Wang, X. Long et al., Rechargeable aqueous zinc-manganese dioxide batteries with high energy and power densities. Nat. Commun. **8**(1), 405 (2017). <https://doi.org/10.1038/s41467-017-00467-x>
23. J. Huang, Z. Wang, M. Hou, X. Dong, Y. Liu et al., Polyaniline-intercalated manganese dioxide nanolayers as a high-performance cathode material for an aqueous zinc-ion battery. Nat. Commun. **9**(1), 2906 (2018). <https://doi.org/10.1038/s41467-018-04949-4>
24. Y. Zhang, Y. Liu, Z. Liu, X. Wu, Y. Wen et al., MnO_2_ cathode materials with the improved stability *via* nitrogen doping for aqueous zinc-ion batteries. J. Energy Chem. **64**, 23–32 (2022). <https://doi.org/10.1016/j.jechem.2021.04.046>
25. D. Kundu, B.D. Adams, V. Duffort, S.H. Vajargah, L.F. Nazar, A high-capacity and long-life aqueous rechargeable zinc battery using a metal oxide intercalation cathode. Nat. Energy **1**, 16119 (2016). <https://doi.org/10.1038/nenergy.2016.119>
26. F. Wan, L. Zhang, X. Dai, X. Wang, Z. Niu et al., Aqueous rechargeable zinc/sodium vanadate batteries with enhanced performance from simultaneous insertion of dual carriers. Nat. Commun. **9**(1), 1656 (2018). <https://doi.org/10.1038/s41467-018-04060-8>
27. C. Liu, Z. Neale, J. Zheng, X. Jia, J. Huang et al., Expanded hydrated vanadate for high-performance aqueous zinc-ion batteries. Energy Environ. Sci. **12**(7), 2273–2285 (2019). <https://doi.org/10.1039/c9ee00956f>
28. J. Ding, Z. Du, L. Gu, B. Li, L. Wang et al., Ultrafast Zn^2+^ intercalation and deintercalation in vanadium dioxide. Adv. Mater. **30**(26), e1800762 (2018). <https://doi.org/10.1002/adma.201800762>
29. M. Yan, P. He, Y. Chen, S. Wang, Q. Wei et al., Water-lubricated intercalation in V_2_ O_5_ ·nH_2_ O for high-capacity and high-rate aqueous rechargeable zinc batteries. Adv. Mater. **30**(1), 1703725 (2018). <https://doi.org/10.1002/adma.201703725>
30. M. Huang, Y. Mai, L. Zhao, X. Liang, Z. Fang et al., Tuning the kinetics of zinc ion in MoS_2_ by polyaniline intercalation. Electrochim. Acta **388**, 138624 (2021). <https://doi.org/10.1016/j.electacta.2021.138624>
31. Z. Zhang, W. Li, R. Wang, H. Li, J. Yan et al., Crystal water assisting MoS_2_ nanoflowers for reversible zinc storage. J. Alloys Compd. **872**, 159599 (2021). <https://doi.org/10.1016/j.jallcom.2021.159599>
32. C. Cai, Z. Tao, Y. Zhu, Y. Tan, A. Wang et al., A nano interlayer spacing and rich defect 1T-MoS_2_ as cathode for superior performance aqueous zinc-ion batteries. Nanoscale Adv. **3**(13), 3780–3787 (2021). <https://doi.org/10.1039/D1NA00166C>
33. T. Jiao, Q. Yang, S. Wu, Z. Wang, D. Chen et al., Binder-free hierarchical VS_2_ electrodes for high-performance aqueous Zn ion batteries towards commercial level mass loading. J. Mater. Chem. A **7**(27), 16330–16338 (2019). <https://doi.org/10.1039/C9TA04798K>
34. J. Liu, W. Peng, Y. Li, F. Zhang, X. Fan, A VS_2_@N-doped carbon hybrid with strong interfacial interaction for high-performance rechargeable aqueous Zn-ion batteries. J. Mater. Chem. C **9**(19), 6308–6315 (2021). <https://doi.org/10.1039/D1TC00531F>
35. T. Chen, X. Zhu, X. Chen, Q. Zhang, Y. Li et al., VS_2_ nanosheets vertically grown on graphene as high-performance cathodes for aqueous zinc-ion batteries. J. Power Sources **477**, 228652 (2020). <https://doi.org/10.1016/j.jpowsour.2020.228652>
